# Inhibition of elastase enhances the adjuvanticity of alum and promotes anti–SARS-CoV-2 systemic and mucosal immunity

**DOI:** 10.1073/pnas.2102435118

**Published:** 2021-08-05

**Authors:** Eunsoo Kim, Zayed Attia, Rachel M. Woodfint, Cong Zeng, Sun Hee Kim, Haley E. Steiner, Rajni Kant Shukla, Namal P. M. Liyanage, Shristi Ghimire, Jianrong Li, Gourapura J. Renukaradhya, Abhay R. Satoskar, Amal O. Amer, Shan-Lu Liu, Estelle Cormet-Boyaka, Prosper N. Boyaka

**Affiliations:** ^a^Department of Veterinary Biosciences, The Ohio State University, Columbus, OH 43210;; ^b^Department of Medicine and Infectious Diseases, University of Sadat City, 32897 Sadat City, Egypt;; ^c^Department of Microbial Immunity and Infection, The Ohio State University, Columbus, OH 43210;; ^d^Infectious Diseases Institute, The Ohio State University, Columbus, OH 43210;; ^e^Food Animal Research Program, Ohio Agricultural Research and Development Center, The Ohio State University, Wooster, OH 44691;; ^f^Department of Pathology, The Ohio State University, Columbus, OH 43210

**Keywords:** mucosal immunity, alum, SARS-CoV-2, elastase, neutrophil elastase inhibitor

## Abstract

Alum, used as an adjuvant in injected vaccines, promotes T helper 2 (Th2) and serum antibody (Ab) responses. However, it fails to induce secretory immunoglobulin (Ig) A (SIgA) in mucosal tissues and is poor in inducing Th1 and cell-mediated immunity. Alum stimulates interleukin 1 (IL-1) and the recruitment of myeloid cells, including neutrophils. We investigated whether neutrophil elastase regulates the adjuvanticity of alum, and whether a strategy targeting neutrophil elastase could improve responses to injected vaccines. Mice coadministered a pharmacological inhibitor of elastase, or lacking elastase, developed high-affinity serum IgG and IgA antibodies after immunization with alum-adsorbed protein vaccines, including the spike protein of severe acute respiratory syndrome coronavirus 2 (SARS-Cov-2). These mice also developed broader antigen-specific CD4^+^ T cell responses, including high Th1 and T follicular helper (Tfh) responses. Interestingly, in the absence of elastase activity, mucosal SIgA responses were induced after systemic immunization with alum as adjuvant. Importantly, lack or suppression of elastase activity enhanced the magnitude of anti–SARS-CoV-2 spike subunit 1 (S1) antibodies, and these antibodies reacted with the same epitopes of spike 1 protein as sera from COVID-19 patients. Therefore, suppression of neutrophil elastase could represent an attractive strategy for improving the efficacy of alum-based injected vaccines for the induction of broad immunity, including mucosal immunity.

## Significance

We report that suppression of the serine protease elastase reshapes innate responses induced by injected vaccines containing alum adjuvant. This reprogramming improves the induction of protective antibodies in the bloodstream and stimulates innate signals, which support the development of antibody responses in mucosal tissues. Our findings identify elastase as the innate regulator that blunts the adjuvant activity of alum. They also demonstrate that vaccination via mucosal routes is not an absolute requirement for antibody responses in mucosal tissues and secretions. Supplementation of an alum-based vaccine containing SARS-CoV-2 spike protein subunit 1 as antigen increased anti–SARS-CoV-2 immunity in the blood and mucosal secretions in mice. Thus, this strategy could help in the development of future protein-based vaccines against SARS-CoV-2.

Aluminum salts or alum used as adjuvant in the majority of injected vaccines promote T helper 2 (Th2) responses and antigen-specific Immunoglobulin (Ig) G (IgG) in the bloodstream, but are poor in inducing cell-mediated immunity. Like other injected vaccines, alum-adsorbed injected vaccines do not promote mucosal immunity, including secretory IgA (SIgA) responses in mucosal tissues (1). The adjuvant activity of alum was originally attributed to a “depot effect” that prolonged the presence of antigen in tissues. This view has been challenged by reports that the site of alum injection can be excised without losing adjuvant activity (2). Alum might not enter into the cells, but rather delivers the adsorbed antigen across the membrane of dendritic cells and enhances the affinity of these cells for CD4^+^ T cells (3). Innate signals stimulated by alum include NLRP3 inflammasome and interleukin 1β (IL-1β) secretion, which activate myeloid cells (i.e., dendritic cells and macrophages) and mast cells (4–8). However, the adjuvant activity of alum also stimulates NLRP3-independent mechanisms (3, 5, 7, 9).

The ability of alum to induce Th2 responses could result from the recruitment of Gr1^+^ cells (i.e., neutrophils) secreting IL-4 (10) and inhibition of IL-12p70 production by dendritic cells (11). Other mechanisms could contribute to the polarization of Th cells by alum. For example, protein-independent engagement of dendritic cell membrane lipids by alum (3), activates the spleen tyrosine kinase (Syk)-phosphinositide-3 kinase (PI3K) pathway (3, 11). Furthermore, alum promotes the production of PGE_2_, a T helper (Th1) suppressor and major IgE inducer, in a Syk- and p38 MAPK-dependent manner (12).

We previously reported an inverse relationship between the recruitment of neutrophils and induction of SIgA after sublingual immunization with a toxin adjuvant (13). We further showed that supplementation with a pharmacological inhibitor of neutrophil elastase (NEI) allowed the development of IgA responses (14). Here, we addressed whether elastase regulates immune responses induced by alum-adsorbed injected vaccines. Our results show that neutrophil elastase limits the breadth of CD4^+^ T cell response and the production of high-affinity serum IgG antibodies. Consistent with this notion, suppression of elastase activity promotes antigen-specific mucosal immunity, including SIgA responses in mice immunized by injection of alum-adsorbed vaccines.

## Results

### Inhibition of Neutrophil Elastase Enhances the Kinetic**s** and Breadth of Serum Antibody Responses Induced by Alum.

**Fig. 1.**
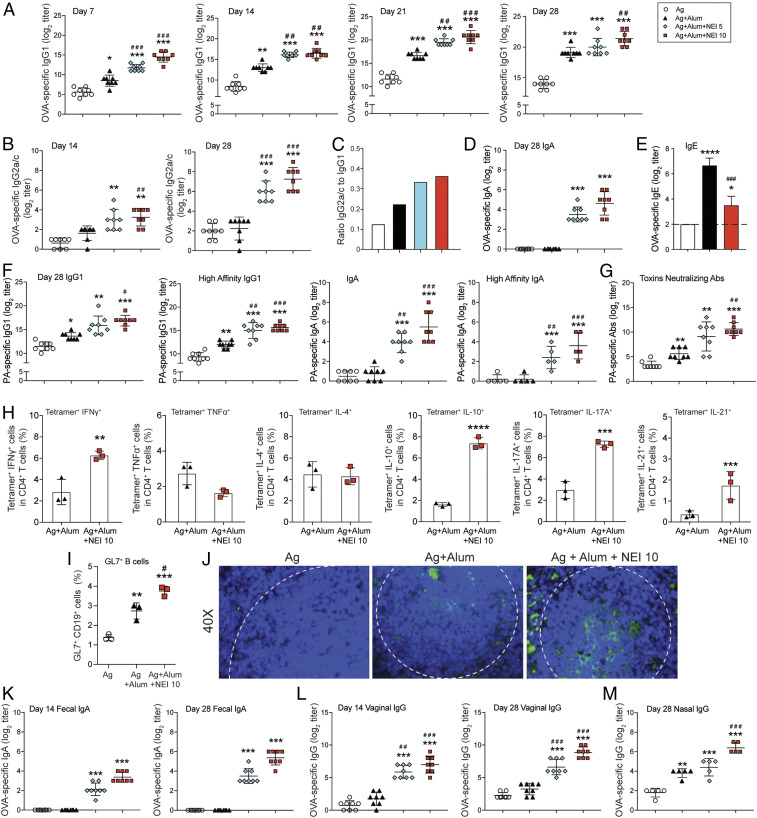
Inhibition of neutrophil elastase enhances the kinetics, breadth, and protective activity of serum Ab responses; broadens the profile of antigen-specific T helper cell responses; and promotes mucosal immunity. Mice were immunized three times, a week apart, by i.p. injection of 100 μL of vaccine containing antigens (Ag) (20 μg PA + 50 μg OVA) alone, Ag adsorbed to alum (2 mg) (Ag + alum), or Ag adsorbed to alum and coadministered with a dose (5 μg or 10 μg) of neutrophil elastase inhibitor (Ag + alum + NEI). OVA- and PA-specific serum Ab responses were analyzed by ELISA. Anti-PA neutralizing Abs were analyzed by the anthrax lethal toxin neutralization assay. (*A*–*E*) OVA-specific responses. (*A*) Time course of IgG1 and (*B*) IgG2a/c responses. (*C*) Ratio of IgG2a/c to IgG1 responses. (*D*) IgA responses. (*E*) IgE responses. (*F* and *G*) PA-specific responses. (*F*) Total and high-affinity IgG1 responses and IgA responses. (*G*) Anti-anthrax toxin-neutralizing Ab titers. (*H*) Antigen-specific T helper cell responses. Spleens were collected on day 28 and restimulated 5 d in vitro with 1 mg/mL of OVA. Cells were then stained with fluorescent-labeled OVA tetramers and fluorescent-labeled anti-CD3, -CD4, -IFNγ, -TNFα, -IL-4, -IL-10, -IL-17A, and -IL-21 and analyzed by flow cytometry. Data are expressed as mean ± SD (representative of three independent experiments with *n* = 3/group). (*I* and *J*) Expression of GL7 and germinal center formation. (*I*) Flow cytometry analysis of GL7^+^ B cells in the spleens on day 14 after first immunization. (*J*) Germinal center formation. Histological section of spleens collected on day 28 after first immunization. Tissues were stained with anti-GL7 and counterstained with DAPI. White dashed lines indicate germinal center (×40) (representative of three independent experiments). (*K*–*M*) Antigen-specific Ab responses in mucosal secretions. (*K*) Fecal extracts, (*L*) vaginal washes, and (*M*) nasal washes were collected at the indicated time points. Antigen-specific IgA and IgG levels were measured by ELISA. Data are expressed as mean Ab titers ± 1 SD (*n* = 5 to 8). *P < 0.05, **P < 0.01, ***P < 0.001, ****P < 0.0001 compared with antigen alone; ^#^*P* < 0.05, ^##^*P* < 0.01, ^###^*P* < 0.005 compared with Ag + alum.

Since alum promotes neutrophil infiltration through stimulation of IL-1α and IL-1β (15), we investigated whether inhibition of neutrophil elastase could regulate antibody (Ab) responses induced by alum. For this purpose, groups of mice were immunized by intraperitoneal injection (i.p.) of a combination of antigens (i.e., ovalbumin [OVA] and *Bacillus anthracis* protective antigen [PA]) either alone, adsorbed to alum as adjuvant (Ag + alum), or Ag + alum supplemented with alvelestat, a highly selective and reversible NEI (Ag + alum + NEI). The adjuvant effect of alum on IgG responses was readily visible 1 wk after the first immunization as mice immunized with Ag + alum showed higher Ag-specific IgG1 titers than mice given Ag alone (Fig. 1 *A*, *Left*). The i.p. route of vaccination was used in this study since mice immunized by i.p. and intramuscular (i.m.) injection of Ag + alum developed the same levels of Ag-specific serum IgG1 responses (*SI Appendix*, Fig. S1*A*). Coadministration of NEI accelerated the kinetics of Ag-specific IgG1 responses in mice immunized with the alum-adsorbed vaccine (Fig. 1*A*). In line with the fact that Th2 responses antagonize IgG2a/c responses in mice (16), antigen-specific IgG2a/c responses were not detected in mice immunized with Ag + alum (Fig. 1*B*). Conversely, NEI supplementation promoted IgG2a/c in a dose-dependent manner (Fig. 1*B*) and increased the IgG2a/IgG1 ratio (Fig. 1*C*), but also Ag-specific IgG3 responses (*SI Appendix*, Fig. S1*B*).

On the other hand, IgA responses could not be detected after immunization with Ag alone or Ag + alum (Fig. 1*D*). In contrast, high levels of serum IgA responses were induced in mice where Ag + alum was supplemented with NEI (Fig. 1*D*). Since mice immunized with Ag alone + NEI did not develop significant IgA responses, these data indicate that NEI lifted a break that prevented alum from promoting broad serum antibody responses. We also found that all Ig isotypes were not increased by NEI as IgE responses were suppressed in animals where the alum-adsorbed vaccine was supplemented with NEI (Fig. 1*E*).

### Coadministration of Neutrophil Elastase Inhibitor Enhances the Affinity and Function of Antibodies Induced by Alum as Adjuvant.

To ensure protection, antibodies need to have sufficient affinity for the antigens and alter their interactions with other ligands. The *B. anthracis* lethal toxin-mediated in vitro toxicity assay provides a functional assay for the assessment of vaccine-induced anti-PA Abs and thus an indication regarding their protection (17, 18). NEI supplementation enhanced the titers of high-affinity PA-specific IgG1 and induced PA-specific serum IgA Abs, which were high-affinity Abs (Fig. 1*F*). In addition, the in vitro cytotoxicity assay showed that NEI supplementation of the alum-adsorbed vaccine enhanced anti-PA neutralizing Abs (Fig. 1*G*).

### Coadministration of Neutrophil Elastase Inhibitor Broadens the Profile of T Helper Responses Induced by Alum as Adjuvant.

Ovalbumin is a well-established model antigen and, unlike *B. anthracis* protective antigen (PA), tetramers are available for analysis of OVA-specific T cell responses. Analysis of both cytokine^+^ CD4^+^ T cell (*SI Appendix*, Fig. S1*C*) and OVA-tetramer^+^ cytokine^+^ CD4^+^ T cell responses (Fig. 1*H*) showed that, when compared with mice immunized with Ag + alum alone, those coadministered NEI developed higher frequencies of Th1 (IFNγ, TNFα), Th2 (IL-4, IL-5), Th17 (IL-17A), and Tfh (IL-21) cells. B cells undergo Ig class switch and somatic hypermutation to increase affinity for antigens in germinal centers. Mice immunized with alum in the presence of NEI exhibited a higher frequency of GL7^+^ B cells (Fig. 1*I*) and larger germinal centers in the spleen (Fig. 1*J*) than mice immunized with Ag alone or Ag + alum, indicating that higher levels of Ig class switch and somatic hypermutation take place in these mice.

### Coadministration of Neutrophil Elastase Inhibitor Promotes SIgA Responses in the Gastrointestinal Tract.

The development of mucosal (oral, nasal, or sublingual) vaccines was primarily driven by the inability of injected vaccines to induce mucosal immunity and SIgA (1, 19). Immunization with neither antigens alone nor Ag + alum induced antigen-specific fecal IgA (Fig. 1*K*). Interestingly, NEI supplementation of Ag + alum vaccine induced antigen-specific fecal SIgA Abs (Fig. 1*K*). NEI supplementation also enhanced the titers of antigen-specific IgG in vaginal (Fig. 1*L*) and nasal washes (Fig. 1*M*). However, coadministration of NEI failed to induce antigen-specific IgA in vaginal or nasal washes, suggesting that NEI selectively promoted homing of IgA-producing cells into the gastrointestinal (GI) tract.

### Neutrophil Elastase Inhibitors Regulate Both Caspase-1–Dependent and –Independent Responses Induced by Alum.

**Fig. 2.**
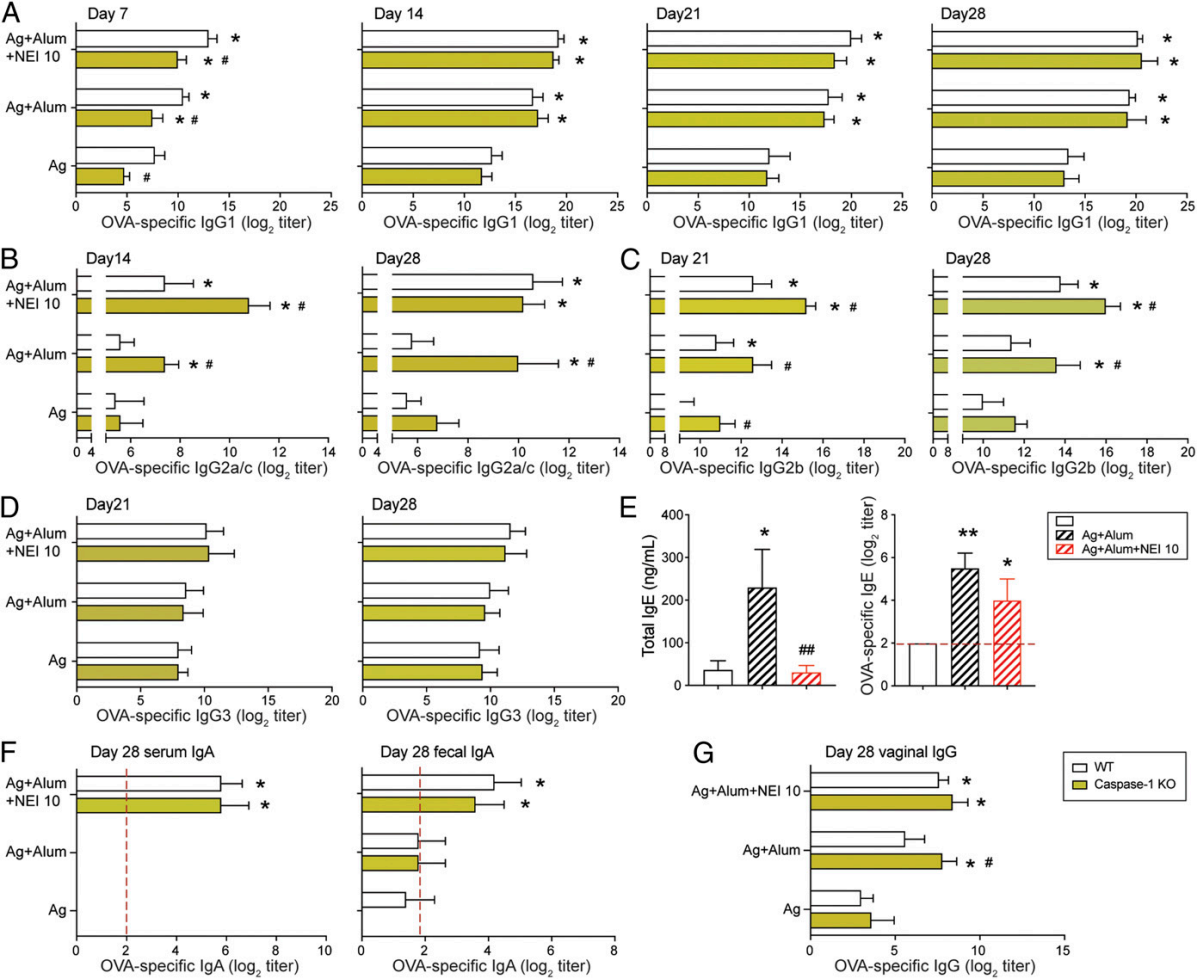
Neutrophil elastase inhibitors regulate both Casp1-dependent and -independent responses induced by alum. Caspase-1 KO and WT C57BL/6 mice were immunized three times, a week apart, by i.p. injection of 100 μL of vaccine containing antigens Ag (50 μg OVA), Ag adsorbed to alum (2 mg) (Ag + alum), or Ag adsorbed to alum and coadministered with 10 μg of neutrophil elastase inhibitor (Ag + alum + NEI 10). OVA-specific serum Ab responses were analyzed by ELISA. (*A*) Time course of OVA-specific IgG1. (*B*) Time course of OVA-specific IgG2a/c responses. (*C*) OVA-specific IgG2b and (*D*) IgG3 responses. (*E*) Total and OVA-specific IgE responses of Caspase-1 KO mice. (*F*) OVA-specific serum and fecal IgA responses. (*G*) OVA-specific IgG responses in vaginal washes. Data are expressed as mean Ab titers ± SD (*n* = 8/group). *P < 0.05, **P < 0.01 compared with antigen alone. ^#^*P* < 0.05, ^##^*P* < 0.01 compared with Ag + alum.

Alum stimulates IL-1β secretion, which activates myeloid cells, including Gr1^+^ cells (4–8, 10). IL-1β can enhance immune responses to vaccines (20) and thus, mediate the adjuvant activity of alum. However, alum also activates mechanisms independent of NLRP3 inflammasome (3, 5, 7, 9). Caspase-1 (Casp1) knockout (KO) mice lack the NLRP3-induced enzyme that catalyzes the cleavage of pro–IL-1β and secretion of active IL-1β (21). One week after immunization with Ag alone or Ag + alum, Casp1 KO developed lower antigen-specific IgG1 responses than control wild-type (WT) mice (Fig. 2*A*). NEI supplementation increased the magnitude of IgG1 responses in both WT and Casp1 KO mice immunized with Ag + alum. By day 14, no difference was observed between the WT and Casp1 KO mice, suggesting that NEI corrected the slower kinetics of IgG1 responses in Casp1 KO mice (Fig. 2*A*). Alum promoted higher IgG2a/c and IgG2b responses in Casp1 KO mice than in control WT mice at day 14 and day 28. These responses were further enhanced by NEI supplementation (Fig. 2 *B* and *C*). No difference was observed between the IgG3 responses in WT and Casp1 KO mice (Fig. 2*D*). In summary, IgG subclass responses induced by alum as adjuvant are differentially regulated by Casp1 and except IgG3, all IgG subclasses were enhanced by NEI supplementation.

Casp1 KO mice developed high IgE titers after immunization with Ag + alum (Fig. 2*E*). Interestingly, supplementation by coadministration of NEI significantly reduced both total and antigen-specific IgE in Casp1 KO mice (Fig. 2*E*). Neither WT nor Casp1 KO mice developed serum IgA or fecal SIgA Abs after immunization with Ag + alum, but they both developed these responses after NEI supplementation (Fig. 2*F*). We found no IgA response in the vaginal washes of WT nor Casp1 KO mice. However, similar levels of antigen-specific IgG responses were measured in the vaginal washes of WT and Casp1 KO mice immunized with Ag + alum + NEI (Fig. 2*G*). Together, these findings show that NEI also regulates NLRP3 inflammasome-independent antibody responses induced by alum as vaccine adjuvant.

### Alum Promotes Broader Serum Antibody Responses in Mice Lacking Neutrophil Elastase.

**Fig. 3.**
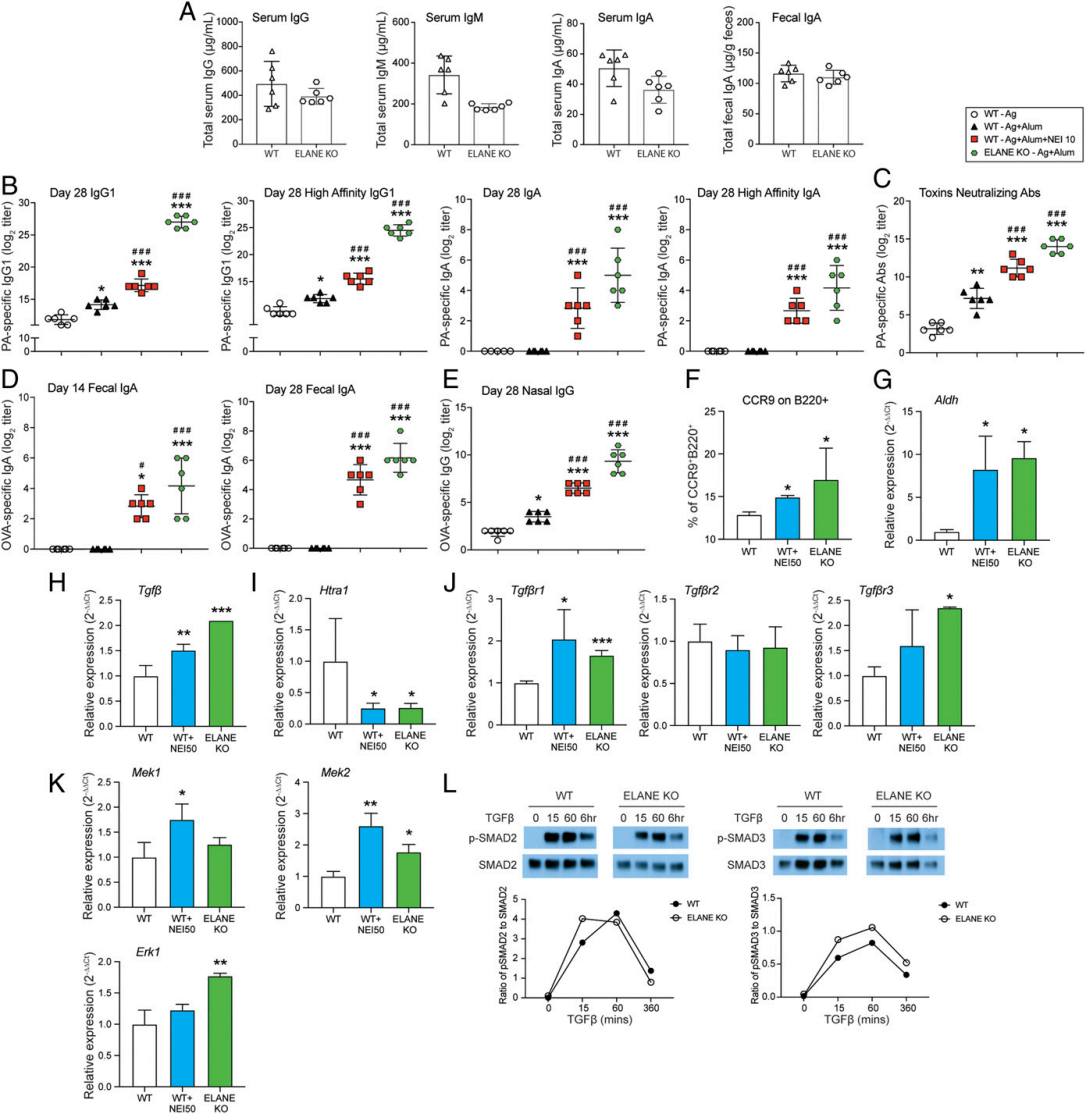
Alum promotes broader serum and mucosal antibody responses in mice lacking neutrophil elastase. (*A*) Basal level of serum IgG, IgM, and IgA and fecal IgA in naïve WT and ELANE KO. (*B*–*E*) ELANE KO mice were immunized three times, a week apart, by i.p. injection of 100 μL of vaccine containing antigens (Ag, 20 μg PA + 50 μg OVA) adsorbed to alum (2 mg) (ELANE KO - Ag + alum). Control WT C57BL/6 mice were immunized with Ag alone (WT Ag), Ag adsorbed to alum (WT Ag + alum), or Ag adsorbed to alum and coadministered with 10 μg of neutrophil elastase inhibitor (Ag + alum + NEI 10). OVA-specific serum Ab responses were analyzed by ELISA. PA-specific serum Ab responses were analyzed by ELISA and by the anthrax lethal toxin neutralizing assay. (*B*) Total and high-affinity PA-specific serum IgG and IgA. (*C*) Anti-anthrax toxin-neutralizing Ab titers. (*D*) Time course of fecal IgA responses. (*E*) Mucosal IgG responses in nasal washes. Data are expressed as mean Ab titers ± SD (*n* = 3 to 6/group). *P < 0.05, **P < 0.01, ***P < 0.001 compared with antigen alone. ^#^*P* < 0.05, ^##^*P* < 0.01, ^###^*P* < 0.001 compared with Ag + alum. (*F*) Expression of CCR9 from B cells. Spleen cells were collected from WT and ELANE KO mice and cultured with NEI (50 μM) for 72 h. (*G*–*K*) Spleen cells were collected from WT and ELANE KO mice and cultured with NEI (50 μM) for 24 h. (*G*) mRNA expression of aldehyde dehydrogenase (*Aldh*). (*H*) mRNA expression of *Tgfβ*. (*I*) mRNA expression of *Htra1*. (*J*) mRNA expression of TGFβ receptors. (*K*) mRNA expression of downstream genes of TGFβ signaling. (*L*) Phosphorylation of SMAD2/3. Spleen cells from WT or ELANE KO mice were collected and starved for 16 h. Cell lysates were harvested after stimulation with TGFβ (5 ng/mL) for 0 min, 15 min, 1 h, or 6 h. Phosphorylated and total SMAD2 and SMAD3 were analyzed by Western blot and Image Studio software was used for quantification of the Western blot bands. *P < 0.05, **P < 0.01, ***P < 0.001 compared with no treatment (WT).

The NEI used in our study is a highly specific inhibitor of elastase with limited activity on other serine proteases. To formally exclude the contribution of an off-target activity of this drug, we analyzed immune responses induced by alum-based injected vaccines in *Elane*^*tm1Sds*^ (ELANE KO) mice that lack neutrophil elastase. Naïve ELANE KO mice contained equivalent levels of IgG, IgM, and IgA in the serum or fecal SIgA than control naïve WT mice (Fig. 3*A*). ELANE KO mice developed higher antigen-specific serum IgG responses to the alum-adsorbed vaccine than WT mice (*SI Appendix*, Fig. S2 *A* and *B*). Interestingly, the kinetics of antigen-specific IgG1 responses of ELANE KO mice was similar to that of WT mice given an alum-based vaccine supplemented with NEI (*SI Appendix*, Fig. S2*A*). ELANE KO mice also developed a broad profile of IgG subclass responses characterized by increased IgG2a/c, IgG2b, and IgG3 (*SI Appendix*, Fig. S2*B*). Antigen-specific serum IgA responses were also induced in ELANE KO mice and they showed similar kinetics and magnitude as those measured in WT mice immunized with Ag + alum + NEI (*SI Appendix*, Fig. S2*C*). Functional characterization of IgG and IgA responses in ELANE KO mice showed that most PA-specific IgG1 and IgA were high affinity antibodies (Fig. 3*B*). In addition, the anthrax toxin-neutralizing activity of PA-specific antibodies was enhanced in ELANE KO mice immunized with Ag + alum and reached the same levels as in WT immunized with Ag + alum + NEI (Fig. 3*C*).

Analysis of mucosal secretions showed that ELANE KO mice immunized with Ag + alum developed mucosal SIgA responses, which were similar to those measured in fecal extract of WT mice immunized with Ag + alum + NEI (Fig. 3*D*). Antigen-specific IgA was not detected in nasal or vaginal washes of ELANE KO mice immunized with Ag (OVA/PA) + alum. However, like WT mice immunized with Ag + alum + NEI, ELANE KO mice developed high titers of antigen-specific IgG in nasal (Fig. 3*E*) and vaginal washes (*SI Appendix*, Fig. S2*D*).

### Elastase Regulates Multiple Pathways That Control Induction of Mucosal Immunity.

As shown above, common features of NEI supplementation and genetic ablation of elastase on host immune response to injected alum-based vaccines are the stimulation of a broader profile of antibody responses, including IgA, and the induction of mucosal immunity after vaccination via a nonmucosal route. Elastase was reported to affect a number of mechanisms that could limit the breadth of immune responses induced by alum as adjuvant. For example, elastase was reported to inhibit the maturation and function of dendritic cells, including the expression of costimulatory molecules (22). Addition of NEI to cultures of spleen cells increased CD86 expression by B cells (*SI Appendix*, Fig. S3*A*). Interestingly, spleen B cells of ELANE KO mice also expressed higher levels of CD40 and CD86 than cells from WT mice (*SI Appendix*, Fig. S4*A*). Both addition of NEI and genetic ablation of elastase increased mRNA levels of *Baff*, pro*Il-1β*, and nerve growth factor (*Ngf*), which are factors that regulate activation and production of antibodies by B cells (*SI Appendix*, Fig. S3*B*). These findings suggest that the increased expression of costimulatory molecules together with increase in BAFF and NGF responses could broaden the profile of antibodies induced by NEI in mice immunized with an alum-based injected vaccine. Furthermore, both the NEI treatment and elastase deficiency increased the expression of the gut homing receptor CCR9 by B cells (Fig. 3*F*). This treatment also increased the transcription of aldehyde dehydrogenase (Fig. 3*G*), a key enzyme needed for production of retinoid acid, which stimulates Ig class switching for production of IgA.

TGFβ signaling is an important player in the induction of IgA and previous reports suggested that serine proteases cleave TGFβ receptors (TGFβR) and thus, antagonize TGFβ signaling (23). Both NEI treatment and lack of elastase lead to increased levels of *Tgfβ* mRNA (Fig. 3*H*). They also reduced levels of mRNA levels of *Htra1* (Fig. 3*I*), an endogenous serine protease reported to cleave TGFβRII and TGFβRIII (23). The latter finding was consistent with the higher levels of *Tgfßr1* and *Tgfßr3* mRNA measured in spleen cells where the elastase activity was reduced or genetically defective (Fig. 3*J*). The mRNA levels of *Mek1*, *Erk1*, and *Mek2* were found to be enhanced in spleen cells treated with NEI or cells from ELANE KO mice, suggesting that the SMAD-independent TGFβ signaling pathway could be regulated by elastase (Fig. 3*K*). Finally, upon in vitro exposure to TGFβ, the kinetics of SMAD2 and SMAD3 phosphorylation was the same in spleen cells of WT and ELANE KO mice (Fig. 3*L*). However, cells from ELANE KO mice achieved higher levels of phospho-SMAD2 (p-SMAD2) at an earlier time point, and their p-SMAD3 levels were higher than in cells from WT mice (Fig. 3*L*).

**Fig. 4.**
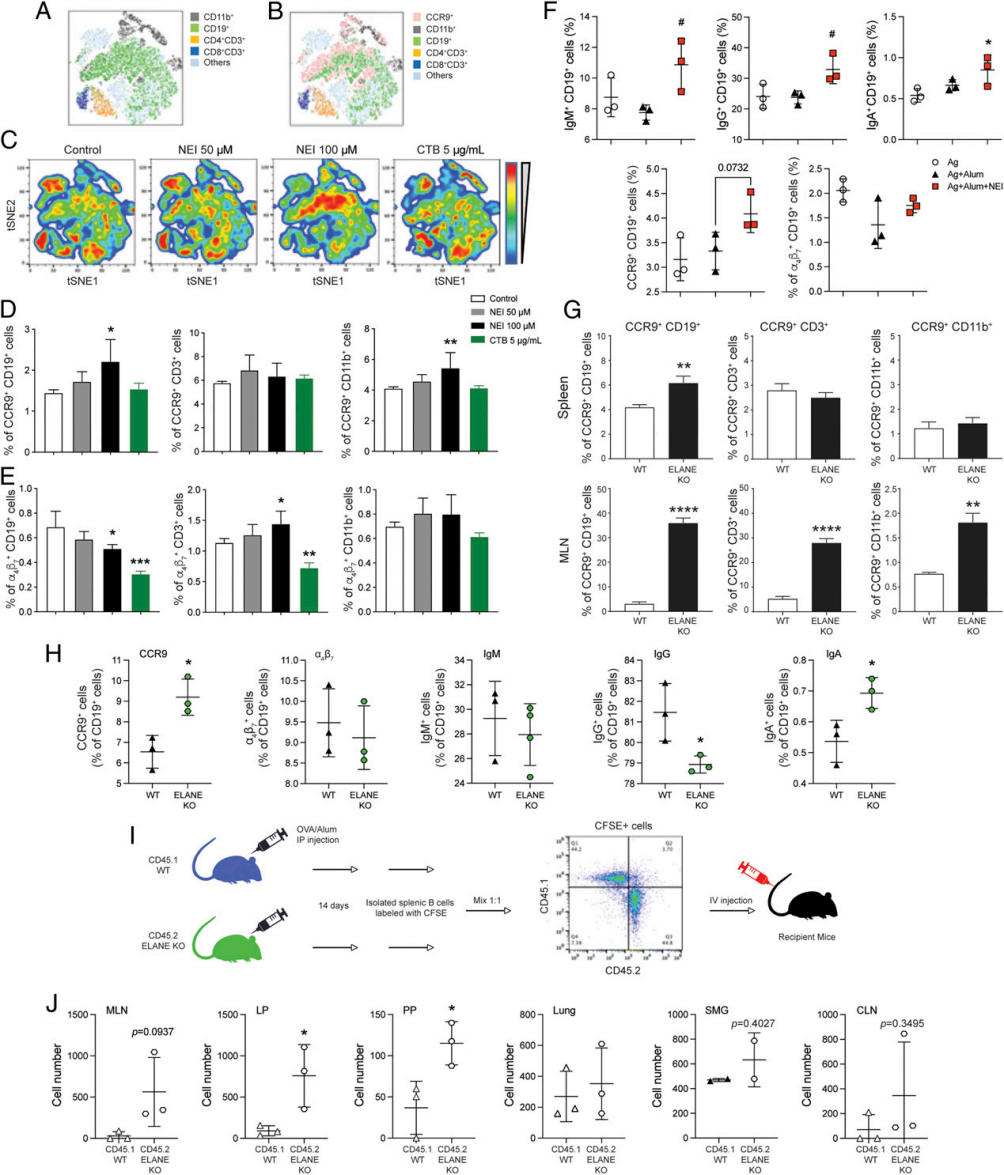
Neutrophil elastase inhibitor or elastase deficiency stimulates the expression of mucosal homing receptors and trafficking of spleen B cells. (*A*–*E*) Spleen cells were cultured for 48 h in the absence (control) or the presence of alvelestat (NEI 50 or 100 μM), or cholera toxin B subunit (CTB; 5 μg/mL). Cells were then stained with fluorescent-labeled antibodies and analyzed by flow cytometry. (*A*) tSNE analysis of cell subsets. (*B*) tSNE analysis of CCR9 and cell subsets. (*C*) tSNE density plots of immune cell profile. (*D* and *E*) Frequency of CCR9^+^ (*D*) and α_4_β_7_^+^ (*E*) in B cells, T cells, and myeloid cells. (*F*) The expression of immunoglobulins and gut homing receptors in spleens after systemic immunization with an alum-based vaccine supplemented with NEI. Spleens were collected on day 14 from mice immunized on days 0 and 7. (*G*) The expression of CCR9 by immune cell subsets after systemic immunization with alum-based vaccine. Mice were immunized by i.p. injection. Spleens and MLNs were collected 48 h later and the expression of CCR9 by B cells (CD19^+^), T cells (CD3^+^), and myeloid cells (CD11b^+^) was analyzed by flow cytometry. (*H*) Frequency of immunoglobulin^+^ spleen B cells and expression of gut homing receptors after systemic immunization of WT and ELANE KO mice with an alum-based vaccine. Spleens were collected on day 14 from mice immunized on days 0 and 7. (*I*) Experimental design for adoptive transfer of purified spleen B cell. CD45.1^+^ WT and CD45.2^+^ ELANE KO mice were immunized with OVA and alum at day 0 and day 7. Splenic B cells were isolated on day 14 and labeled with CFSE. CD45.1^+^ and CD45.2^+^ B cells were mixed with 1:1 ratio and transferred into naïve WT recipient mice. (*J*) The number of CFSE^+^ cells in tissues including MLNs, LP, PPs, lung, SMGs, and CLNs were measured by flow cytometry. Data are expressed as mean ± SD (*n* = 3 to 6/group). *P < 0.05, **P < 0.01, ***P < 0.001, ****P < 0.0001 compared with the control group; ^#^*P* < 0.05 compared with Ag + alum.

The best-described factors regulating homing of immune cells into the gut are CCR9 and α4β7 (1, 24). In this regard, addition of NEI to cultures of spleen cells enhanced the expression of CCR9 by B lymphocytes (CD19^+^) and myeloid cells (CD11b^+^), while α4β7 expression was increased in T cells (Fig. 4 *A*–*E*). Furthermore, 2 wk (day 14) after initial immunization, the spleens of mice immunized with the alum-based vaccine supplemented with NEI contained a high frequency of immunoglobulin^+^ B cells that expressed CCR9 (Fig. 4*F*). These results are consistent with the fact that, 48 h after injection of the alum-based vaccine, ELANE KO mice contained higher numbers of B cells and T cells expressing CCR9 in the spleen, and mesenteric lymph nodes (MLNs) than WT mice (Fig. 4*G*). In order to conclusively demonstrate that suppression or lack of elastase promoted the mucosal homing of effector cells, spleen B cells isolated from WT (CD45.1) or ELANE KO (CD45.2) mice immunized 2 wk earlier with Ag + alum were adoptively transferred into naïve recipient WT mice (Fig. 4 *H*–*J*). Flow cytometry analysis of spleen cells before transfer showed a higher frequency of CCR9^+^ B cells and IgA^+^ B cells in the spleens of ELANE KO mice (Fig. 4*H*). Tracking of purified splenic B cells transferred to naïve recipient mice showed that 18 h after the transfer, B cells from ELANE KO mice more efficiently populated the mucosal tissues of the gut (i.e., intestinal lamina propria [LP] and Peyer’s patches [PPs]) than B cells from WT mice (Fig. 4*J*). The cells from ELANE KO mice also exhibited a trend toward increased tropism for mucosal tissues of the airways (cervical lymph nodes [CLNs] and submandibular glands [SMGs]) (Fig. 4*J*).

We also tested whether NEI could regulate immune factors in other animal species. We previously showed that NEI stimulates the expression of BAFF and IL-10 and secretion of IgG and IgA by murine spleen cells, in vitro (25). Addition of NEI to cultures of pig spleen cells stimulated IgG and IgA secretion (*SI Appendix*, Fig. S3*C*). Finally, to determine whether NEI could also regulate human B responses, peripheral blood mononuclear cells (PBMCs) from healthy donors were incubated with NEI. NEI enhanced the expression of *BAFF* and *IL-10* and costimulatory molecules (i.e., *CD40* and *CD80*) by human PBMCs, in vitro (*SI Appendix*, Fig. S3*D*). Together, these findings suggest that NEI could improve immune responses to vaccines in other species, including humans.

### Suppression of Elastase Improves Anti–SARS-CoV-2 Systemic and Mucosal Responses to an Alum-Adsorbed Injected Vaccine.

**Fig. 5.**
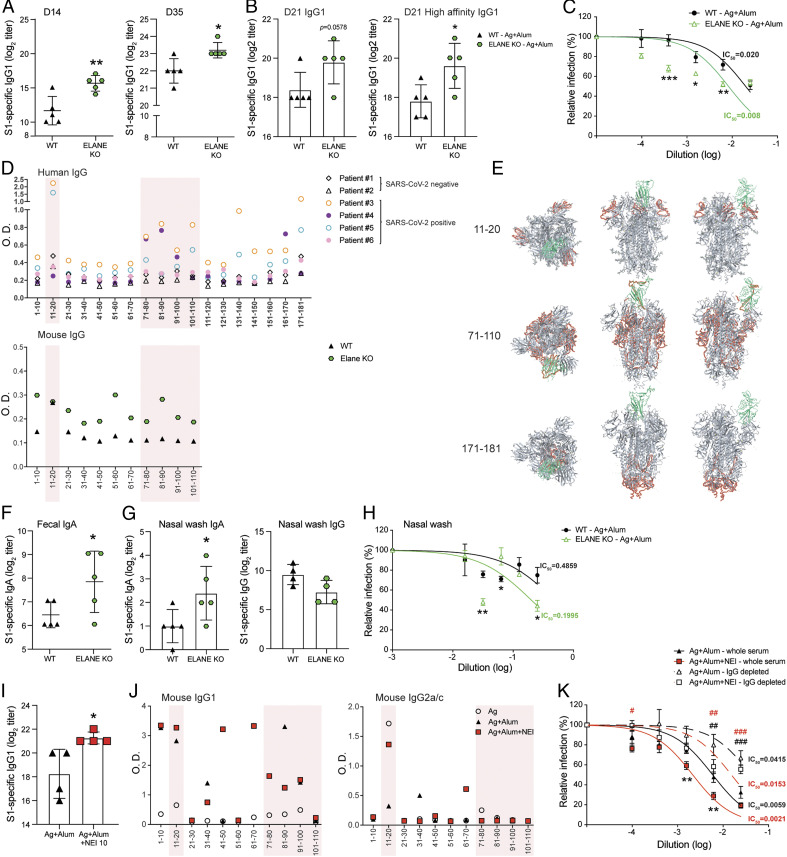
Lack of neutrophil elastase enhances antibody responses and neutralization activity against immunization of SARS-CoV-2. ELANE KO mice and WT mice were immunized three times, a week apart, by intraperitoneal injection of 100 μL of vaccine containing antigens (10 μg PA + 20 μg S1 of SARS-CoV-2) adsorbed to alum (2 mg) (Ag + alum). (*A* and *B*) S1-specific serum Ab responses were analyzed by ELISA. (*A*) Time course of S1-specific serum IgG1 responses. (*B*) Total and high-affinity S1-specific serum IgG1. (*C*) Pseudovirus neutralization assay. SARS-CoV-2 pseudovirus was incubated with dilutions of serum samples before infection of HEK293T/ACE2 cells and calculation of IC_50_. (*D*) B cell epitope mapping of antibodies in sera from patients and immunized mouse. Sera of patients and immunized mice at 1:500 dilution were analyzed by IgG ELISA with pooled peptides of spike protein of SARS-CoV-2. (*E*) Top and side views of 3D imaging of spike protein and localization of epitopes of IgG. Epitopes are indicated in red. Receptor binding domain (RBD) is shown in green. (*F* and *G*) S1-specific mucosal IgA or IgG responses. (*F*) S1-specific fecal IgA, (*G*) nasal wash IgA and IgG. (*H*) Pseudovirus neutralization assay with nasal wash samples. (*I*–*K*) WT mice were immunized three times, a week apart, by intraperitoneal injection of 100 μL of vaccine containing antigens (Ag, 10 μg PA + 20 μg S1 of SARS-CoV-2) and Ag adsorbed to alum (2 mg) (Ag + alum), or Ag adsorbed to alum and coadministered with 10 μg of neutrophil elastase inhibitor (Ag + alum + NEI 10). (*I*) S1-specific serum IgG1 responses. (*J*) Epitope mapping from immunized mice. Sera of immunized mice at 1:250 dilution were analyzed by IgG1 or IgG2a/c ELISA with pooled peptides. (*K*) Pseudovirus neutralization assay. Sera were collected on day 21 and depleted of IgG. Several dilutions of whole serum and IgG-depleted serum were added to SARS-CoV-2 pseudovirus and IC_50_ was calculated. Data are expressed as mean ± 1 SD. (*n* = 4 to 5/group). *P < 0.05, **P < 0.01 compared with Ag + alum. ^#^*P* < 0.05, ^##^*P* < 0.01, ^###^*P* < 0.001 IgG-depleted compared with whole serum.

The spike (S) protein on SARS-CoV-2 virions is a promising candidate for a vaccine as it is: 1) indispensable for viral entry into the host cells through binding to angiotensin-converting enzyme 2 (ACE2) expressed on the host cells, and 2) the major target of virus neutralizing antibodies in patients who recover from COVID-19. While the airway is the primary site of SARS-CoV-2 infection, ACE2 is also expressed by intestinal epithelial cells and cells in other extrapulmonary locations (26, 27). We examined whether suppression of elastase could represent a strategy for inducing protective immunity by injected alum-based SARS-CoV-2 vaccines. For this purpose, mice were immunized with recombinant S1 protein and alum as adjuvant. ELANE KO mice developed higher titers of S1 protein-specific serum IgG (Fig. 5*A*), which were also high-affinity IgG antibodies (Fig. 5*B*). Most importantly, the neutralizing antibody titer of sera from ELANE KO mice was significantly higher than that of WT mice immunized with the same injected alum-based SARS-CoV-2 vaccine (Fig. 5*C*).

Next, we evaluate how the reactivity of anti-S1 antibodies induced by vaccination of mice compare to those of patients exposed to SARS-CoV-2. Sera of convalescent COVID-19 patients exhibited high reactivity against pools of B cell epitopes corresponding to the N-terminal (peptides 1 to 20), central (peptides 71 to 110), and C-terminal (peptides 171 to 181) portion of the S protein. Sera of WT mice immunized with the adsorbed SARS-CoV-2 S1 protein vaccine primarily reacted with the pool of peptides corresponding to the N-terminal (peptides 11 to 20) portion of the S1 molecule. Interestingly, sera of ELANE KO mice immunized with the adsorbed SARS-CoV-2 S1 protein vaccine reacted with the same B cell epitopes recognized by sera from convalescent COVID-19 patients (Fig. 5 *D* and *E*). This finding suggests that the broadening of IgG subclass response in ELANE KO mice allowed alum-based vaccination with SARS-CoV-2 S1 protein to generate serum antibodies with similar reactivity to S1 protein than after infection. In contrast with WT mice, ELANE KO mice immunized with the alum-adsorbed SARS-CoV-2 S1 protein developed S1 protein-specific IgA responses in the GI tract (i.e., fecal extracts) (Fig. 5*F*) and respiratory tract (i.e., nasal washes) (Fig. 5*G*). Furthermore, the pseudovirus neutralization activity of nasal washes was significantly higher in samples from ELANE KO mice (Fig. 5*H*).

In order to conclusively demonstrate that NEI supplementation of alum-based vaccine can increase the level of protection against SARS-CoV-2 infection we analyzed anti–SARS-CoV-2 S1 protein in WT mice immunized with Ag + alum or Ag + alum + NEI. Consistent with our finding with ELANE KO mice, NEI supplementation increased the magnitude of serum anti-S1 protein antibodies (Fig. 5*I*). Interestingly, NEI supplementation increased the breadth of B cell epitopes recognized by IgG1 and IgG2a/c (Fig. 5*J* and *SI Appendix*, Fig. S4). Finally, we compared the protective activity of whole sera and IgG-depleted sera to establish the role of IgA in protection against virus infection. Sera of mice immunized with a vaccine supplemented with NEI exhibited a significantly greater neutralization activity than those of mice given the vaccine alone in the pseudovirus neutralizing assay (Fig. 5*K*). While depletion of IgG reduced neutralization activity, sera of mice immunized with a vaccine supplemented with NEI retained a significant level of neutralizing activity, which was higher than that measured after IgG depletion in sera from mice immunized with the vaccine without NEI (Fig. 5*K*). Collectively, our findings suggest that NEI supplementation can improve the efficacy of alum-adsorbed anti–SARS-CoV-2 vaccines, including by promoting the induction of IgA in the serum and mucosal secretions.

## Discussion

Aluminum salts (alum) are extensively used as adjuvants in injected vaccines. They promote Th2 responses that support IgG responses in the bloodstream, but are poor inducers of Th1 and cell-mediated immunity. Like most injected vaccines, alum-based vaccines do not induce immunity in mucosal tissues, including SIgA, which could provide a layer of protection against entry of infectious agents in the respiratory, enteric, or urogenital tracts. Using a pharmacological inhibitor of elastase and mice lacking elastase, we show that elastase is a major regulator of the adjuvant activity of alum. Specifically, elastase activity slows down the kinetics of antibody responses, blunts the profile of T helper cell responses, and prevents the induction of mucosal IgA responses by injected alum-adsorbed vaccines.

Neutrophils contribute to host defense through secretion of neutrophil extracellular traps (NETs) and release of primary/azurophilic granules, which contain defensins, myeloperoxidase, lysozymes, and three serine proteases: neutrophil elastase, cathepsin G, and protease 3 (28, 29). But, unlike dendritic cells and macrophages (1, 30), neutrophils have not been extensively studied for their role in bridging innate and adaptive immunity. Elastase was shown to inhibit the maturation and function of dendritic cells, including expression of CD40, CD80, and CD86 (22), and neutrophil depletion was reported to enhance T helper and IgG responses to vaccination (31). Our findings that neutrophil elastase is a key regulator of immune responses to alum-based injection vaccines are in line with the report that the regulatory effect of neutrophils does not require physical contact with dendritic cells or T cells (31). They also support the notion that a product(s) secreted by neutrophils can regulate both the magnitude and quality of adaptive immunity. In this regard, human neutrophil peptide defensins were shown to enhance serum IgG responses to nasally coadministered antigens (32). Here we show that neutrophil elastase plays a central role in the development of mucosal immunity and SIgA responses.

It is striking that a temporary inhibition of elastase activity was sufficient to accelerate the kinetics of serum IgG responses to injected alum-adsorbed vaccine. This point has significant public health implications as it suggests that NEI supplementation can speed up induction of immunity by alum-adsorbed vaccines. NEI induction of antibody-stimulating cytokines BAFF and IL-10 (*SI Appendix*, Fig. S3) (25) could be the accelerating factor. NEI supplementation or lack of elastase could facilitate the migration of vaccine-primed cells, as suggested by others who showed that depletion of neutrophils favors the development of T responses at distant lymph nodes (33). Increased mobility of vaccine-primed immune cells in mice immunized with an NEI-supplemented vaccine could help increase the titers of high-affinity antibodies. In this regard, neutrophil depletion during the early stage of lupus accelerated germinal center formation (34). Here we show that NEI supplementation increases the size of germinal centers. Nonetheless, this did not result in a polyclonal enhancement of all Ig isotypes since it suppressed IgE responses. Considering that induction of IgE is a potential safety risk, NEI supplementation represents an approach to increase the safety of vaccines.

The main immunological appeal of mucosal (oral or nasal) vaccines is their ability to induce mucosal SIgA (1, 19). We recently showed that NEI supplementation is a realistic substitute to neutrophil depletion as an approach to induce mucosal IgA by sublingual vaccines (14). To our knowledge, this is evidence that the adjuvanticity of alum can be modulated to allow the development of mucosal immunity and more specifically antigen-specific SIgA in mucosal tissues and secretions. We have previously shown that NEI stimulates expression of IgA-promoting cytokines IL-10 and TGFβ by murine spleen cells (25). The fact that NEI stimulates expression of these cytokines by human PBMCs (*SI Appendix*, Fig. S3*D*) as well as IgA production by pig spleen cells (*SI Appendix*, Fig. S3*C*), suggests that NEI promotes a microenvironment that reshapes the innate responses induced by alum to help production of mucosal IgA in gut tissues. It is striking that although NEI supplementation increased IgG responses in mucosal tissues of the airway and genitourinary tracts, SIgA was only induced in the GI tract of mice immunized with the alum-based vaccine. Retinoic acid (RA) is a vitamin A metabolite synthesized from precursor molecules such as retinol. It is now well established that RA is highly expressed by dendritic cells and macrophages in gut-associated lymphoid tissues and that it stimulates the expression of gut homing receptors (35). Furthermore, RA stimulates the generation of gut-homing IgA-secreting B cells (36). Thus, we hypothesize that NEI supplementation or lack of elastase facilitated the homing of vaccine-primed B cells to the gut where mucosal antigen presenting cells further provide RA signals for differentiation into gut-homing IgA-secreting B cells.

In summary, this work shows that elastase, the major product of neutrophil granules, plays a key role in shaping the immune responses to alum-based injected vaccines. Specifically, suppression of elastase activity accelerates serum IgG responses and broadens the profile of T helper responses. Furthermore, NEI promotes the development of mucosal immunity including SIgA, and this has important implications for the development of future vaccines for protection against pathogens that infect one or several mucosal sites. This notion is supported by our finding that NEI supplementation broadens the antibody responses to an alum-adsorbed anti–SARS-CoV-2 vaccine, including by promoting IgA in the serum and mucosal secretions.

## Materials and Methods

### Animals.

Specific pathogen-free (SPF) wild-type C57BL/6J mice and Elane^tm1Sds^ (ELANE KO) mice, which lack elastase, were obtained from The Jackson Laboratory. Casp1 KO mice were obtained from Vishva M. Dixit, Genentech, San Francisco, CA. All mice were maintained at the Ohio State University (OSU) animal care facility and provided food and drink ad libitum. Porcine spleens were specimens from White-Duroc crossbred pigs raised in the BSL2 facility at the Ohio Agricultural Research and Development Center (OARDC) and used as controls for other unrelated studies. All animal experiments were approved by the OSU Animal Care and Use Committee.

### Patient Samples and Specimens.

All samples were deidentified specimens from a clinical laboratory, and handling of these samples was under an approved institutional review board (IRB) protocol (OSU 2020H0228). Plasma and serum were collected from hospitalized COVID-19 inpatients or intensive care unit (ICU) patients, OSU health care workers, and blinded convalescent plasma donors and analyzed in a blinded manner.

### Immunization.

Mice were sensitized three times, at weekly intervals, by i.p. or i.m. injection of 100 μL of saline containing vaccine antigens (50 μg of OVA [Sigma-Aldrich] plus 20 μg of PA [*B. anthracis* protective antigen, BEI Resources]) or 50 μg of recombinant SARS-CoV-2 spike protein S1 subunit (Val16-Gln690) (RayBiotech). Mice were vaccinated with the antigen(s) alone (Ag) or vaccine antigens adsorbed on alum (aluminum hydroxide and magnesium hydroxide, Imject, Thermo Fisher Scientific) (Ag + alum). To address the effect of NEI supplementation, groups of mice were injected with alum-adsorbed vaccine antigens plus different doses (5 μg or 10 μg) of the neutrophil inhibitor alvelestat (AZD9668, C_25_H_22_F_3_N_5_O_4_S) (Selleckchem) (Ag + alum + NEI). Mice that received the NEI showed no change in their vitality, food consumption, and body weight.

### Evaluation of Antigen-Specific Antibody Responses.

To determine OVA-specific and PA-specific antibody titers, an ELISA was performed as described previously (13, 17, 37, 38). For assessment of IgA responses in the intestinal secretions, freshly emitted fecal pellets were normalized by homogenization in phosphate buffered saline (1 mL per 0.1 g feces). After centrifugation, dilutions of supernatants were used for evaluation of antigen-specific IgA levels as described above.

### Analysis of Total and Antigen-Specific Serum IgE Ab Responses.

Total IgE Ab levels were determined by a BD OptEIA Set Mouse IgE, (BD PharMingen) according to instructions from the manufacturer. To prevent interference of IgG in the assay, serial dilutions of immune plasma were previously depleted of IgG by overnight incubation in Reacti-Bind Protein G coated plates (Pierce) (38). Antigen-specific IgE was detected with a biotinylated anti-mouse IgE Ab (BD Biosciences).

### Quantification of High-Affinity Antibody Responses.

High-affinity antibody responses were measured by ELISA as described above after addition of urea (4 mM) to remove antibodies bound to the antigen with low affinity (14, 39).

### Assessment of Toxin-Neutralizing Antibodies.

A toxin-neutralization assay was performed as previously described (13, 14, 17, 18). Briefly, sample dilutions were added to J774 macrophages cultured in RPMI 1640 media supplemented with 10% fetal calf serum. *B. anthracis* lethal toxin (LeTx) (i.e., PA plus *B. anthracis* lethal factor [LF] [List Biological]) was then added to the plates. After overnight incubation, MTT [3-(4,5-dimethylthiazol-2-yl)-2,5-diphenyl tetrazolium bromide; Sigma-Aldrich] was added to assess the viability of macrophages as a function of redox potential. The toxin-neutralizing antibody titers were determined as the lowest concentration of serum that protects macrophages from the cytotoxicity of LeTx.

### Immunohistochemistry.

Tissues were formalin fixed and paraffin embedded. Sections (5 µm thick) were stained with anti-GL7 (clone: GL7, dilution 1:100) (BioLegend), and nuclei were counterstained with DAPI (4',6-diamidino-2-phenylindole).

### In Vivo Trafficking of B Cells.

To identify the mucosal sites of B cell trafficking, we performed adoptive cell transfer. Briefly, CD45.1 WT and CD45.2 ELANE KO mice were immunized with Ag (OVA) and alum on days 0 and 7. Spleens were collected on day 14 and B cells were isolated (EasySep Mouse B cell isolation kit, Stemcell Technologies) and stained with CFSE (5 μM carboxyfluorescein succinimidyl ester, BioLegend). For adoptive transfer, 10^7^ cells of a 1:1 mixture of CFSE-stained CD45.1 and CD45.2 B cells were administered to wild-type (CD45.1) mice by tail vein injection. Recipient mice were killed 18 h later and CFSE^+^ cells present in the spleens or mucosal tissues were analyzed by flow cytometry.

### B Cell Epitope Mapping.

An array of 181 peptides of 17- or 13-mers, with 10 amino acid overlaps that span the spike (S) glycoprotein of the USA-WA1/2020 (GenPept: QHO60594) of SARS-CoV-2 (NR-52402, BEI Bioresources) were used to identify linear B cell epitopes recognized by anti-SARS spike protein S1 antibodies. Briefly, microtiter plates were coated with individual peptide (20 μg/mL). Samples were then added and the binding antibodies were detected with horseradish peroxidase (HRP)-conjugated anti-mouse γ-specific antisera or biotin-conjugated rat anti-mouse IgG1 or IgG2a/c followed by HRP-conjugated streptavidin. For identification of linear B cell epitopes recognized by antibodies present in the sera of COVID-19 patients, the binding antibodies were detected with HRP-conjugated anti-human IgG. To show the location of epitopes on protein, epitopes were labeled on three-dimensional (3D) structure of SARS-CoV-2 spike protein (Protein Data Bank [PDB] ID: 6ZOW) by using an iCn3D web-based 3D structure viewer provided by the National Center for Biotechnology Information (NCBI).

### Analysis of Antigen-Specific T Helper Cell Cytokine Responses and Expression of Homing Receptors.

Antigen-specific T helper cell cytokine responses were analyzed by flow cytometry after in vitro restimulation and intracellular staining with cytokine-specific fluorescent antibodies. Expression of the gut homing receptors CCR9 and α4β7 was analyzed by flow cytometry as staining with anti-CCR9 and anti-α4β7 antibodies (BioLegend).

### In Vitro Culture of Immune Cells with NEI.

Murine spleen cells were cultured for 48 h in the presence of NEI doses (50 or 100 μM). Control cells were cultured in the absence of effector (control) or in the presence of cholera toxin B subunit (5 μg/mL). Cells were then analyzed by flow cytometry. Human PBMCs were cultured for 48 h in the absence (control) or presence of various doses of NEI, and cytokine and costimulatory molecule mRNA was analyzed by real-time RT-PCR. Pig spleen cells were cultured for 6 d with various doses of NEI.

### Real-Time RT-PCR.

Tissues were collected, snap frozen, and reduced to powder before adding TRIzol (Invitrogen). Complementary DNA was synthesized using SuperScript III (Invitrogen). Real-time RT-PCR was performed as previously described (37) using the primers listed in *SI Appendix*.

Data were expressed as relative mRNA expression = 2^-ΔΔCt^ where ΔCt = Ct_unknown_ − Ct_HKG_, and normalized against the housekeeping gene (β-actin).

### Quantification of Porcine IgM, IgG, and IgA.

Porcine IgM, IgG, and IgA were measured using an ELISA using immunoglobulin standards and anti-pig IgM, IgG, and IgG antibodies (Bio-Rad).

### SARS-CoV-2 Pseudovirus Neutralization Assay.

For determination of virus neutralizing activity, lentiviral SARS-CoV-2 pseudotyped virus was constructed and used as described in the previous study (40). Specifically, 100 μL of virus were incubated with sera or nasal wash solutions for 1 h at 37 °C and the mixture was added to HEK293T/ACE2 cells preseeded in 96-well plates. Gluc or Nluc activity was measured at 72 h after infection for viral infectivity. For luciferase measurement, 20 μL of supernatant was collected from each well and transferred to a white nonsterile 96-well plate, and 20 μL of Gluc substrate (0.1 M Tris [Millipore Sigma, T6066] pH 7.4, 0.3 M sodium ascorbate [Spectrum, S1349], 10 μM coelenterazine [GoldBio, CZ2.5]) was added. Luminescence was immediately read by a plate reader. In order to establish the relative contribution of IgG compared with other Ig isotypes, in selected experiments, IgG in the samples were depleted with the aid of anti-mouse IgG MicroBeads (Miltenyi) prior to the virus neutralization assay.

### Statistical Analysis.

Results are expressed as the mean ± 1 SD. Statistical significance was determined by one-way ANOVA, followed by Tukey’s post hoc test. All statistical analyses were performed with StataSE 12.0 software (StataCorp LLC) and Prism 7 software (GraphPad Software).

## Supplementary Material

Supplementary File

## Data Availability

All study data are included in the article and/or *SI Appendix*.

## References

[1] P. N. Boyaka, Inducing mucosal IgA: A challenge for vaccine adjuvants and delivery systems. J. Immunol. 199, 9–16 (2017). 28630108 10.4049/jimmunol.1601775 PMC5719502

[2] S. Hutchison., Antigen depot is not required for alum adjuvanticity. FASEB J. 26, 1272–1279 (2012). 22106367 10.1096/fj.11-184556 PMC3289510

[3] T. L. Flach., Alum interaction with dendritic cell membrane lipids is essential for its adjuvanticity. Nat. Med. 17, 479–487 (2011). 21399646 10.1038/nm.2306

[4] S. C. Eisenbarth, O. R. Colegio, W. O’Connor, F. S. Sutterwala, R. A. Flavell, Crucial role for the Nalp3 inflammasome in the immunostimulatory properties of aluminium adjuvants. Nature 453, 1122–1126 (2008). 18496530 10.1038/nature06939 PMC4804622

[5] L. Franchi, G. Núñez, The Nlrp3 inflammasome is critical for aluminium hydroxide-mediated IL-1beta secretion but dispensable for adjuvant activity. Eur. J. Immunol. 38, 2085–2089 (2008). 18624356 10.1002/eji.200838549 PMC2759997

[6] V. Hornung., Silica crystals and aluminum salts activate the NALP3 inflammasome through phagosomal destabilization. Nat. Immunol. 9, 847–856 (2008). 18604214 10.1038/ni.1631 PMC2834784

[7] M. Kool., Cutting edge: Alum adjuvant stimulates inflammatory dendritic cells through activation of the NALP3 inflammasome. J. Immunol. 181, 3755–3759 (2008). 18768827 10.4049/jimmunol.181.6.3755

[8] F. A. Sharp., Uptake of particulate vaccine adjuvants by dendritic cells activates the NALP3 inflammasome. Proc. Natl. Acad. Sci. U.S.A. 106, 870–875 (2009). 19139407 10.1073/pnas.0804897106 PMC2630092

[9] A. S. McKee., Alum induces innate immune responses through macrophage and mast cell sensors, but these sensors are not required for alum to act as an adjuvant for specific immunity. J. Immunol. 183, 4403–4414 (2009). 19734227 10.4049/jimmunol.0900164 PMC2912728

[10] M. B. Jordan, D. M. Mills, J. Kappler, P. Marrack, J. C. Cambier, Promotion of B cell immune responses via an alum-induced myeloid cell population. Science 304, 1808–1810 (2004). 15205534 10.1126/science.1089926

[11] A. Mori., The vaccine adjuvant alum inhibits IL-12 by promoting PI3 kinase signaling while chitosan does not inhibit IL-12 and enhances Th1 and Th17 responses. Eur. J. Immunol. 42, 2709–2719 (2012). 22777876 10.1002/eji.201242372

[12] E. Kuroda., Silica crystals and aluminum salts regulate the production of prostaglandin in macrophages via NALP3 inflammasome-independent mechanisms. Immunity 34, 514–526 (2011). 21497116 10.1016/j.immuni.2011.03.019

[13] J. Jee., Neutrophils negatively regulate induction of mucosal IgA responses after sublingual immunization. Mucosal Immunol. 8, 735–745 (2015). 25563500 10.1038/mi.2014.105 PMC4481173

[14] J. C. Rowe, Z. Attia, E. Kim, E. Cormet-Boyaka, P. N. Boyaka, A novel supplementation approach to enhance host response to sublingual vaccination. Sci. Rep. 9, 715 (2019). 30679470 10.1038/s41598-018-36370-8 PMC6346055

[15] E. Oleszycka., IL-1α and inflammasome-independent IL-1β promote neutrophil infiltration following alum vaccination. FEBS J. 283, 9–24 (2016). 26536497 10.1111/febs.13546

[16] F. D. Finkelman., Lymphokine control of in vivo immunoglobulin isotype selection. Annu. Rev. Immunol. 8, 303–333 (1990). 1693082 10.1146/annurev.iy.08.040190.001511

[17] A. Duverger., Contributions of edema factor and protective antigen to the induction of protective immunity by *Bacillus anthracis* edema toxin as an intranasal adjuvant. J. Immunol. 185, 5943–5952 (2010). 20952678 10.4049/jimmunol.0902795 PMC4053574

[18] A. Duverger., *Bacillus anthracis* edema toxin acts as an adjuvant for mucosal immune responses to nasally administered vaccine antigens. J. Immunol. 176, 1776–1783 (2006). 16424208 10.4049/jimmunol.176.3.1776

[19] P. N. Boyaka, K. Fujihashi, “Host defense at mucosal surfaces” in Clinical Immunology. Principles and Practice, R. R. Rich., Eds. (Elsevier, 2019), pp. 285–298.

[20] H. F. Staats, F. A. Ennis Jr, IL-1 is an effective adjuvant for mucosal and systemic immune responses when coadministered with protein immunogens. J. Immunol. 162, 6141–6147 (1999). 10229857

[21] S. Christgen, D. E. Place, T. D. Kanneganti, Toward targeting inflammasomes: Insights into their regulation and activation. Cell Res. 30, 315–327 (2020). 32152420 10.1038/s41422-020-0295-8 PMC7118104

[22] A. Roghanian, E. M. Drost, W. MacNee, S. E. Howie, J. M. Sallenave, Inflammatory lung secretions inhibit dendritic cell maturation and function via neutrophil elastase. Am. J. Respir. Crit. Care Med. 174, 1189–1198 (2006). 16959917 10.1164/rccm.200605-632OC

[23] J. R. Graham., Serine protease HTRA1 antagonizes transforming growth factor-β signaling by cleaving its receptors and loss of HTRA1 in vivo enhances bone formation. PLoS One 8, e74094 (2013). 24040176 10.1371/journal.pone.0074094 PMC3770692

[24] J. R. Mora, U. H. von Andrian, Differentiation and homing of IgA-secreting cells. Mucosal Immunol. 1, 96–109 (2008). 19079167 10.1038/mi.2007.14

[25] Z. Attia., Inhibitors of elastase stimulate murine B lymphocyte differentiation into IgG- and IgA-producing cells. Eur. J. Immunol. 48, 1295–1301 10.1002/eji.201747264. (2018). 29710424 PMC6447050

[26] F. Hikmet., The protein expression profile of ACE2 in human tissues. Mol. Syst. Biol. 16, e9610 (2020). 32715618 10.15252/msb.20209610 PMC7383091

[27] C. G. K. Ziegler., SARS-CoV-2 receptor ACE2 is an interferon-stimulated gene in human airway epithelial cells and is detected in specific cell subsets across tissues. Cell 181, 1016–1035.e19 (2020). 32413319 10.1016/j.cell.2020.04.035 PMC7252096

[28] B. Amulic, C. Cazalet, G. L. Hayes, K. D. Metzler, A. Zychlinsky, Neutrophil function: From mechanisms to disease. Annu. Rev. Immunol. 30, 459–489 (2012). 22224774 10.1146/annurev-immunol-020711-074942

[29] N. Borregaard, O. E. Sørensen, K. Theilgaard-Mönch, Neutrophil granules: A library of innate immunity proteins. Trends Immunol. 28, 340–345 (2007). 17627888 10.1016/j.it.2007.06.002

[30] A. Iwasaki, R. Medzhitov, Regulation of adaptive immunity by the innate immune system. Science 327, 291–295 (2010). 20075244 10.1126/science.1183021 PMC3645875

[31] C. W. Yang, B. S. Strong, M. J. Miller, E. R. Unanue, Neutrophils influence the level of antigen presentation during the immune response to protein antigens in adjuvants. J. Immunol. 185, 2927–2934 (2010). 20679530 10.4049/jimmunol.1001289 PMC3509756

[32] J. W. Lillard Jr, P. N. Boyaka, O. Chertov, J. J. Oppenheim, J. R. McGhee, Mechanisms for induction of acquired host immunity by neutrophil peptide defensins. Proc. Natl. Acad. Sci. U.S.A. 96, 651–656 (1999). 9892688 10.1073/pnas.96.2.651 PMC15191

[33] C. W. Yang, E. R. Unanue, Neutrophils control the magnitude and spread of the immune response in a thromboxane A2-mediated process. J. Exp. Med. 210, 375–387 (2013). 23337807 10.1084/jem.20122183 PMC3570104

[34] A. K. Bird., Neutrophils slow disease progression in murine lupus via modulation of autoreactive germinal centers. J. Immunol. 199, 458–466 (2017). 28584005 10.4049/jimmunol.1700354 PMC5524201

[35] M. Iwata., Retinoic acid imprints gut-homing specificity on T cells. Immunity 21, 527–538 (2004). 15485630 10.1016/j.immuni.2004.08.011

[36] J. R. Mora., Generation of gut-homing IgA-secreting B cells by intestinal dendritic cells. Science 314, 1157–1160 (2006). 17110582 10.1126/science.1132742

[37] A. Bonnegarde-Bernard., IKKβ in intestinal epithelial cells regulates allergen-specific IgA and allergic inflammation at distant mucosal sites. Mucosal Immunol. 7, 257–267 (2014). 23839064 10.1038/mi.2013.43 PMC4053573

[38] E. Kim., Intestinal epithelial cells regulate gut eotaxin responses and severity of allergy. Front. Immunol. 9, 1692 (2018). 30123215 10.3389/fimmu.2018.01692 PMC6085436

[39] K. H. Chan., Use of antibody avidity assays for diagnosis of severe acute respiratory syndrome coronavirus infection. Clin. Vaccine Immunol. 14, 1433–1436 (2007). 17881505 10.1128/CVI.00056-07 PMC2168165

[40] C. Zeng., Neutralizing antibody against SARS-CoV-2 spike in COVID-19 patients, health care workers, and convalescent plasma donors. JCI Insight 5, e143213 (2020). 10.1172/jci.insight.143213 PMC7710271 33035201

