# Supplementary material for: Inhibition of elastase enhances the adjuvanticity of alum and promotes anti–SARS-CoV-2 systemic and mucosal immunity
Source: Proc Natl Acad Sci U S A. 2021 Aug 5;118(34):e2102435118. doi: 10.1073/pnas.2102435118 (PMC8403952; doi:10.1073/pnas.2102435118)
Supplement: Supplementary File [file pnas.2102435118.sapp.pdf]

SUPPLEMENTAL INFORMATION

Inhibition of elastase enhances the adjuvanticity of alum and promotes anti-SARS-CoV-2 systemic and mucosal immunity

Eunsoo Kim[a,1], Zayed Attia[a,b,1,2], Rachel M. Woodfint[a], Cong Zeng[a], Sun Hee Kim[a], Haley E. Steiner[a], Rajni Kant Shukla[a], Namal P.M. Liyanage[a,c,d], Shristi Ghimire[e], Jianrong Li[a,d], Gourapura J. Renukaradhya[e], Abhay R. Satoskar[d,f], Amal O. Amer[c,d], Shan-Lu Liu[a,c,d], Estelle Cormet-Boyaka[a,d], and Prosper N. Boyaka[a,c,d3]

[a]*Dept. of Veterinary Biosciences, The Ohio State University, Columbus, OH 43210*

[b]*Dept of Medicine and Infectious Diseases, University of Sadat City,Sadat City 32897, Egypt*

[c]*Dept. Microbial Immunity and Infection, The Ohio State University, Columbus, OH 43210*

[d]*Infection Diseases Institute, The Ohio State University, Columbus, OH 43210*

[e]*Food animal research program, Ohio Agricultural Research and Development Center, The Ohio State University, Wooster, OH 44691*

[f]*Dept. of Pathology, The Ohio State University, Columbus, OH 43210*

[1]These authors contributed equally to this work.

[2]Current address: The Vaccine and Immunity Center, Abigail Wexner Research Institute, Nationwide Children's Hospital, Columbus, OH 43205.

[3]**Address correspondence**: Dr. Prosper N. Boyaka, The Ohio State University, 1900 Coffey Road, VMAB 354, Columbus, OH 43210. Email: boyaka.1@osu.edu

Keywords: Alum, elastase, neutrophil elastase inhibitor, mucosal immunity, IgA, SARS-CoV-2

EXTENDED MATERIALS AND METHODS

Animals

Specific pathogen-free (SPF) wild-type C57BL/6J mice and Elane $^{tm1Sds/}$ (ELANE KO) mice which lack elastase were obtained from Jackson Laboratory (Bar Harbor, ME). Caspase 1 KO mice were obtained from (Dr. Dixit, Genentech). All mice were maintained at the Ohio State University animal care facility and were provided food and drink ad libitum. Porcine spleens were specimens from White-Duroc crossbred pigs raised in the BSL2 facility at OARDC and used as controls for other unrelated studies. All animal experiments were approved by the OSU Animal Care and Use Committee.

Patient samples and specimens.

All samples were deidentified specimens from a clinical laboratory, and handling of these samples was under an approved IRB protocol (OSU 2020H0228). Plasma and serum were collected from hospitalized COVID-19 inpatients or ICU patients, OSU HCWs, and blinded convalescent plasma donors and analyzed in a blinded manner.

Immunization

Mice were sensitized three times, at weekly intervals, by i.p. or i.m. injection of 100 □l of saline containing vaccine antigens [50 µg of OVA (Sigma-Aldrich, Saint Louis, MO) plus 20 µg of PA (*Bacillus anthracis* protective antigen, BEI Resources, Manassas, VA)] or 50 µg of recombinant SARS-CoV-2 Spike protein S1 subunit (Val16-Gln690) (RayBiotech, Peachtree Corners, GA). Mice were vaccinated with the antigen(s) alone (Ag) or vaccine antigens adsorbed on alum (aluminum hydroxide and magnesium hydroxide, Imject™, ThermoFisher Scientific, Waltham, MA) (Ag+Alum). To address the effect of NEI supplementation, groups of mice were injected

with alum-adsorbed vaccine antigens plus different doses (5 μg or 10 μg) of the neutrophil inhibitor Alvelestat (AZD9668, $C_{25}H_{22}F_3N_5O_4S$) (Selleckchem, Houston, TX) (Ag+Alum-NEI). Mice that received the NEI showed no change in their vitality, food consumption and body weight. Blood, fecal and vaginal wash samples were collected weekly, and nasal washes were collected at the time of euthanasia to monitor serum and mucosal antibody immunoglobulin isotypes and subclass responses.

Evaluation of antigen-specific antibody responses

To determine OVA-specific and PA-specific antibody titers, ELISA was performed as described previously (1-4). Briefly, microtiter plates were coated with OVA (1 mg/ml) or PA (5 μg/ml). For detection of OVA-specific IgG and IgA Abs, serum or fecal material extracts were serially diluted in PBS 1% BSA, added to the plates and the binding antibodies were detected with HRP-conjugated anti-mouse γ- or α-heavy chain-specific antisera (Southern Biotech Associates Inc., Birmingham, AL). Biotin-conjugated rat anti-mouse IgG1, IgG2a/c, IgG2b or IgG3 monoclonal Abs and HRP-conjugated streptavidin (BD Bioscience, san Jose, CA) were used to measure IgG subclass responses. The reactions were revealed by addition of the water-soluble HRP substrate ABTS (2,2'-Azinobis [3-ethylbenzothiazoline-6-sulfonic acid]-diammonium salt, Sigma-Aldrich) and the Ab titers were determined as the last dilutions of samples with an absorbance of > 0.1 above that of control samples from naïve mice.

For assessment of IgA responses in the intestinal secretions, freshly emitted fecal pellets were normalized by homogenization in PBS (1 ml per 0.1 g feces). After centrifugation, dilutions of supernatants were used for evaluation of antigen-specific IgA levels as described above.

Analysis of total and antigen-specific serum IgE Ab responses.

Total IgE Ab levels were determined by a BD OptEIA Set Mouse IgE, (BD PharMingen) according to instructions from the manufacturer. To prevent interference of IgG in the assay, serial dilutions of immune plasma were previously depleted of IgG by overnight incubation in Reacti-Bind Protein G Coated Plates (Pierce, Rockford, IL) (4). In order to detect antigen-specific IgE, the microtiter plates were coated with OVA (1 mg/mL) or PA (5 μg/ml). Serial dilutions of IgG-depleted plasma were then added and IgE were detected with a biotinylated anti-mouse IgE Ab (BD Biosciences). The IgE titers were determined as described above for IgG and IgA.

Quantification of high affinity antibody responses

High affinity antibody responses were measured by ELISA as described above with a minor modification. Briefly, plates were coated with PA and incubated with dilutions of the samples. Urea (4 mM) was then added and the plates incubated for 30 min at room temperature to remove antibodies that bound to the antigen with low affinity (5, 6). After washing, the detection antibodies were added and the remaining steps of the ELISA conducted as described above.

Assessment of toxin neutralizing antibodies

Toxin neutralization assay was performed as previously described (2, 3, 6, 7). Briefly, sample dilutions were added to J774 macrophages cultured in cultured in RPMI supplemented with 10 % fetal calf serum. *Bacillus anthracis* lethal toxin (LeTx) [i.e., PA plus *Bacillus anthracis* lethal factor (LF, List Biological, Campbell, CA)] was then added to the plates. After overnight incubation, MTT (3-(4,5-dimethylthiazol-2-yl)-2,5-diphenyl tetrazolium bromide; Sigma-Aldrich) was added to assess the viability of macrophages as a function of redox potential. The toxin

neutralizing antibody titers were determined as the lowest concentration of serum that protects macrophages from the cytotoxicity of LeTx.

Immunohistochemistry.

Tissues were formalin-fixed and paraffin-embedded. Sections (5 µm thick) were stained anti-GL7 (clone: GL7, dilution 1:100) (Biolegend), and nuclei were counterstained with DAPI.

***In vivo* trafficking of B cells**

To identify the mucosal sites of B cell trafficking, we performed adoptive cell transfer. Briefly, CD45.1 WT and CD45.2 ELANE KO mice were immunized with Ag (OVA) and alum on days 0 and 7. Spleens were collected on day 14 and B cells were isolated (EasySep Mouse B cell isolation Kit, Stemcell technologies, Vancouver, Canada) and stained with CFSE (5µM carboxyfluorescein succinimidyl ester, Biolegend). For adoptive transfer, 10^7 cells of a 1:1 mixture of CFSE-stained CD45.1 and CD45.2 B cells was administered to wild-type (CD45.1) mice by tail vein injection. Recipient mice were euthanized 18hr later and $CFSE^+$ cells present in the spleens or mucosal tissues were analyzed by flow cytometry.

B cell epitope mapping

Array of 181-peptides of 17- or 13-mers, with 10 amino acid overlaps that span the spike (S) glycoprotein of the USA-WA1/2020 (GenPept: QHO60594) of the SARS-CoV-2 (NR-52402, BEI Bioresources) was used to identify linear B cell epitopes recognized by anti-SARS Spike protein S1 antibodies. Briefly, microtiter plates were coated with individual peptide (20 µg/ml). Samples were then added and the binding antibodies were detected with HRP-conjugated anti-mouse γ-

specific antisera or biotin-conjugated rat anti-mouse IgG1 or IgG2a/c followed by HRP-conjugated streptavidin. For identification of linear B cell epitopes recognized antibody in the sera of COVID-19 patients, the binding antibodies were detected with HRP-conjugated anti-human IgG. To show the location of epitopes on protein, epitopes were labeled on 3D structure of SARS-CoV-2 spike protein (PDB ID: 6ZOW) by using iCn3D web-based 3D structure viewer provided from NCBI.

Analysis of antigen-specific T helper cell cytokine responses and expression of homing receptors

Antigen-specific T helper cell cytokine responses were analyzed by flow cytometry after *in vitro* restimulation and intracellular staining with cytokine-specific fluorescent antibodies. Briefly, splenocytes and mesenteric lymph nodes were collected on day 28 after the first immunization and restimulated with antigen (i.e., 1 mg/ml of OVA or 15 μg/ml of PA) *in vitro* as previously described (2, 3, 6, 7). After 5 days culture, cells were subjected to extracellular staining with lineage-specific antibodies [i.e., anti-CD3, and anti-CD4 (Biolegend, San Diego, CA)] and OVA tetramer (I-A^b chicken Ova 328-337, NIH Tetramer Core Facility). Cells were then fixed and subjected to intracellular staining using Th1 (IFNγ, TNFα), Th2 (IL-4, IL-5, IL-10), Th17 (IL-17A), and Tfh (IL-21) cytokine-specific antibodies (Biolegend). Flow cytometry analysis was performed with an Attune NxT flow cytometer (Thermo Fisher Scientific, Waltham, MA).

Expression of the gut homing receptors CCR9 and α4β7 was analyzed by flow cytometry as staining with anti-CCR9 and anti-α4β7 antibodies (Biolegend). The t-Distributed Stochastic Neighbor Embedding (t-SNE) analysis of CCR9 was performed using FlowJo (v10, BD, Ashland, OR) with 550 of iteration, 30 of perplexity, and 630 of learning rate. To allow comparison, samples (n=3 per group) were down sampled to 3000 events and concatenated into a single fcs file

providing identical t-SNE map distribution. Cell subset density from each groups were expressed as pseudocolors.

***In vitro* culture of immune cells with NEI**

Murine spleen cells, pig spleen cells and human PBMC were used to assess the effect of NEI on immune cells, *in vitro.* Murine spleen cells were cultured for 48 hrs in the presence of NEI doses (50 or 100 μM). Control cells were cultured in the absence of effector (control) or in the presence of cholera toxin B subunit (5 μg/ml). Cells were then analyzed by flow cytometry. Human PBMC were cultured for 48 hrs in the absence (control) or presence of various doses of NEI and cytokine and costimulatory molecule mRNA analyzed by real-time RT-PCR. Pig spleen cells were cultured for 6 days with various doses of NEI.

Real-time RT- PCR.

Tissues were collected, snap frozen, and reduced to powder before adding TRIzol (Invitrogen, Carlsbad, CA). Complementary DNA was synthesized using Superscript III (Invitrogen). Real-time RT-PCR was performed as previously described (1) using the following primers:

hβ-ACTIN forward TGG GCA TGG GTC AGA AGG AT; *hβ-ACTIN* reverse GCT CGA TGG GGT ACT TCA GG; *hBAFF* forward AGC AGA AAT AAG CGT GCC GT; *hBAFF* reverse TGG TGT TTC ACT GTC TGC AA; *hIL-10* forward GGC ACC CAG TCT GAG AAC AG; *hIL-10* reverse ACT CTG CTG AAG GCA TCT CG; *hCD40* forward AAA AGG TGG CCA AGA AGC CA; *hCD40 reverse* TGT TGG AGC CAG GAA GAT CG; *hCD80* forward GCA GGG AAC ATC ACC ATC CA ; *hCD80* reverse ACG TGG ATA ACA CCT GAA CAG A. *mβ-actin* forward GCG CAA GTA CTC TGT GTG GA; *mβ-actin* reverse GAA AGG GTG TAA AAC

GCA GC; *mTgfβ forward* CCC TAT ATT TGG AGC CTG GA; *mTgfβ reverse* CTT GCG ACC CAC GTA GTA GA; *mTgfβr1 forward* TGC CAT AAC CGC ACT GTC A; *mTgfβr1 reverse* AAT GAA AGG GCG ATC TAG TGA TG; *mTgfβr2 forward* AGC ATC ACG GCC ATC TGT G; *mTgfβr2 reverse* TGG CAA ACC GTC TCC AGA GT; *mTgfβr3 forward* TTG CGG AGT ACC TTC AAC CC; *mTgfβr3 reverse* AGG ATT GGA GTT GGG GGA GA; *mBaff* forward AGG CTG GAA GAA GGA GAT GAG; *mBaff* reverse CAG AGA AGA CGA GGG AAG GG; *mIl-1β* forward TCG CAG CAG CAC ATC AAC AAG; *mIl-1β* reverse CCA GCA GGT TAT CAT CAT CAT CC; *mAsc* forward GAC AGT ACC AGG CAG TTC GT; *mAsc* reverse AGT CCT TGC AGG TCA GGT TC; *mIl-6* forward CCG GAG AGG AGA CTT CAC AG; *mIl-6* reverse TCC ACG ATT TCC CAG AGA AC; *mNgf* forward TGA CTT TGG AGC TGG CCT TAT; *mNgf* reverse AGG CAC AGC ATG TTC ACT AGG; *mAldh* forward CGC AAG CTC TCT GTA ACT CCG TCA; *mAldh* reverse CAA CAC CTG GGG AAC AGA GCA GC; *mHtra1* forward CAA AGA GCT GGG ACT TCG GA; *mHtra1* reverse CAC CTC GCC ATC CAG GTT TA; *mMek1* forward TTT CTC CAA GCT GGG GCT G; *mMek1* reverse GAA CTC TCG CCT CAG CAC A; *mMek2* forward TCC CAC CTA TCC CAC CAG TC; *mMek2* reverse CTG AGA GGG GCA GCA AGA AG; *mErk1* forward CTT CAA CCC AAA CAA GCG CA; *mErk1* reverse CAG CTC CAT GTC GAA GGT GA. Data were expressed as relative mRNA expression = $2^{-\Delta\Delta Ct}$ where $\Delta Ct = Ct_{unknown} - Ct_{HKG}$, and normalized against the house-keeping gene (β-ACTIN).

Quantification of porcine IgM, IgG, and IgA.

Porcine IgM, IgG, and IgA were measured using an ELISA using immunoglobulin standards and anti-pig IgM, IgG, and IgG antibodies (Bio-Rad).

SARS-CoV-2 pseudovirus neutralization assay.

For determination of virus neutralizing activity, lentiviral SARS-CoV-2 pseudotyped virus was constructed and used as described in the previous study (8). Specifically, 100 μL of virus were incubated with sera or nasal wash solutions for 1 h at 37C and the mixture was added to HEK293T/ACE2 cells pre-seeded in 96-well plates. Gluc or Nluc activity was measured at 72 hours after infection for viral infectivity. For luciferase measurement, 20 μL of supernatant were collected from each well and transferred to a white nonsterile 96-well plate, and 20 μL of Gluc substrate (0.1M Tris [MilliporeSigma, T6066] pH 7.4, 0.3M sodium ascorbate [Spectrum, S1349], 10 μM coelenterazine [GoldBio, CZ2.5]) was added. Luminescence was immediately read by a plate reader. In order to establish the relative contribution of IgG compared to other Ig isotypes, in selected experiments, IgG in the samples were depleted with the aid of anti-mouse IgG MicroBeads (Miltenyi) prior to the virus neutralization assay.

Statistical analysis

Results are expressed as the mean ± 1 one standard deviation. Statistical significance was determined by one-way ANOVA, followed by Tukey post-hoc test. All statistical analyses were performed with the StataSE 12.0 software (StataCorp LLC, College Station, TX) and Prism 7 software (Graphpad Software, La Jolla, CA).

REFERENCES

1. A. Bonnegarde-Bernard *et al.*, IKKbeta in intestinal epithelial cells regulates allergen-specific IgA and allergic inflammation at distant mucosal sites. *Mucosal Immunol* **7**, 257-267 (2014).
2. A. Duverger *et al.*, Contributions of edema factor and protective antigen to the induction of protective immunity by Bacillus anthracis edema toxin as an intranasal adjuvant. *J Immunol* **185**, 5943-5952 (2010).
3. J. Jee *et al.*, Neutrophils negatively regulate induction of mucosal IgA responses after sublingual immunization. *Mucosal Immunol* **8**, 735-745 (2015).
4. E. Kim *et al.*, Intestinal Epithelial Cells Regulate Gut Eotaxin Responses and Severity of Allergy. *Front Immunol* **9**, 1692 (2018).
5. K. H. Chan *et al.*, Use of antibody avidity assays for diagnosis of severe acute respiratory syndrome coronavirus infection. *Clin Vaccine Immunol* **14**, 1433-1436 (2007).
6. J. C. Rowe, Z. Attia, E. Kim, E. Cormet-Boyaka, P. N. Boyaka, A Novel Supplementation Approach to Enhance Host Response to Sublingual Vaccination. *Sci Rep* **9**, 715 (2019).
7. A. Duverger *et al.*, Bacillus anthracis edema toxin acts as an adjuvant for mucosal immune responses to nasally administered vaccine antigens. *J Immunol* **176**, 1776-1783 (2006).
8. C. Zeng *et al.*, Neutralizing antibody against SARS-CoV-2 spike in COVID-19 patients, health care workers, and convalescent plasma donors. *JCI Insight* **5,** e143213 (2020).

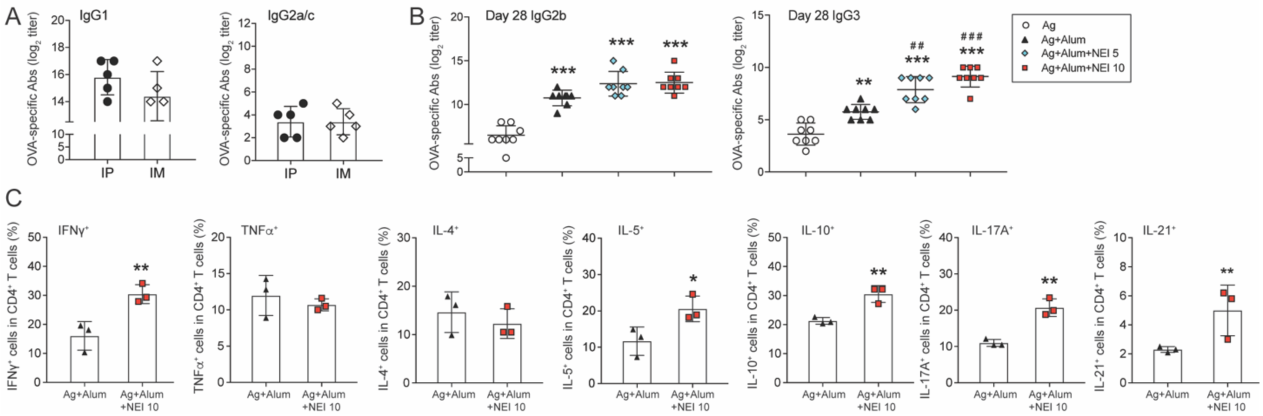

Figure S1. Co-administration of neutrophil elastase inhibitor enhances the kinetic and breadth of serum Ab responses and broadens the profile of T helper cell responses induced by alum as adjuvant. Mice were immunized three times, a week apart, by intraperitoneal injection (i.p) of 100 µl of vaccine containing antigens (Ag) (20 µg PA + 50 µg OVA) alone, Ag adsorbed to alum (2 mg) (Ag+Alum), or Ag adsorbed to alum and co-administered with a dose (5 µg or 10 µg) of neutrophil elastase inhibitor (Ag+Alum+NEI). Control mice were immunized by intramuscular (i.m.) injection of 100 µl of vaccine containing antigens Ag adsorbed to alum (2 mg) (Ag+Alum). OVA-specific serum Ab responses were analyzed by ELISA. (A) Comparison of IgG1 responses (day 14) after im or ip immunization. (B) OVA-specific serum IgG2b and IgG3 responses two weeks after the last immunization (day 28). (C) OVA-specific $CD4^+$ T cell cytokine responses. $CD4^+$ T cell cytokine responses analyzed by flow cytometry after in vitro re-stimulation. Data are expressed as mean Ab titers ± SD. (n=5-8/group). $*p < 0.05$, $**p < 0.01$, $***p < 0.001$ compared to antigen alone. $^{###}p < 0.001$ compared to Ag+Alum.

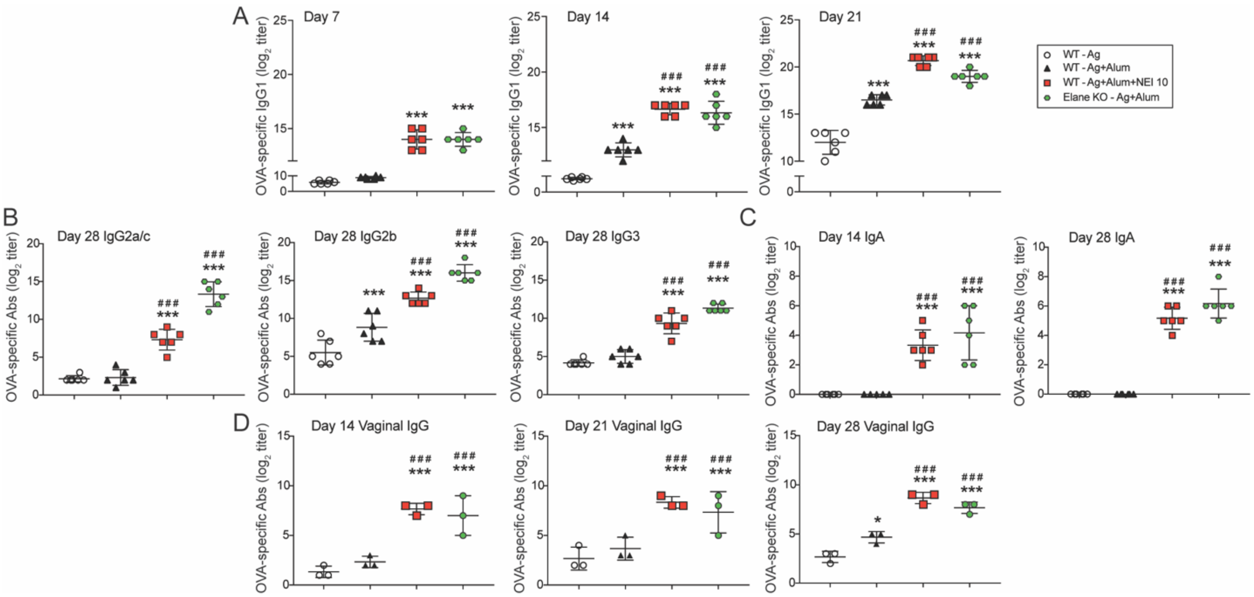

Figure S2. Mice lack of neutrophil elastase develop broader antibody responses to injected vaccines with alum as adjuvant. ELANE KO mice were immunized three times, a week apart, by i.p. injection of 100 µl of vaccine containing antigens (Ag, 20 µg PA + 50 µg OVA) adsorbed to alum (2 mg) (ELANE KO - Ag+Alum). Control wild-type C57BL/6 mice were immunized with Ag alone (WT Ag), Ag adsorbed to alum (WT Ag+Alum), or Ag adsorbed to alum and co-administered with 10 µg of neutrophil elastase inhibitor (Ag+Alum+NEI 10). OVA-specific serum Ab responses were analyzed by ELISA. PA-specific serum Ab responses were analyzed by ELISA and by the anthrax lethal toxin toxicity assay. (A) Time course of OVA-specific serum IgG1 responses. (B) OVA-specific serum IgG subclass responses two weeks after the last immunization (day 28). (C) Time course of OVA-specific serum IgA responses. (D) Time course of OVA-specific vaginal IgG. $*p < 0.05$, $**p < 0.01$, $***p < 0.001$ compared to the control group. $^{\#}p < 0.05$, $^{\#\#}p < 0.01$, $^{\#\#\#}p < 0.001$ compared to Ag+Alum.

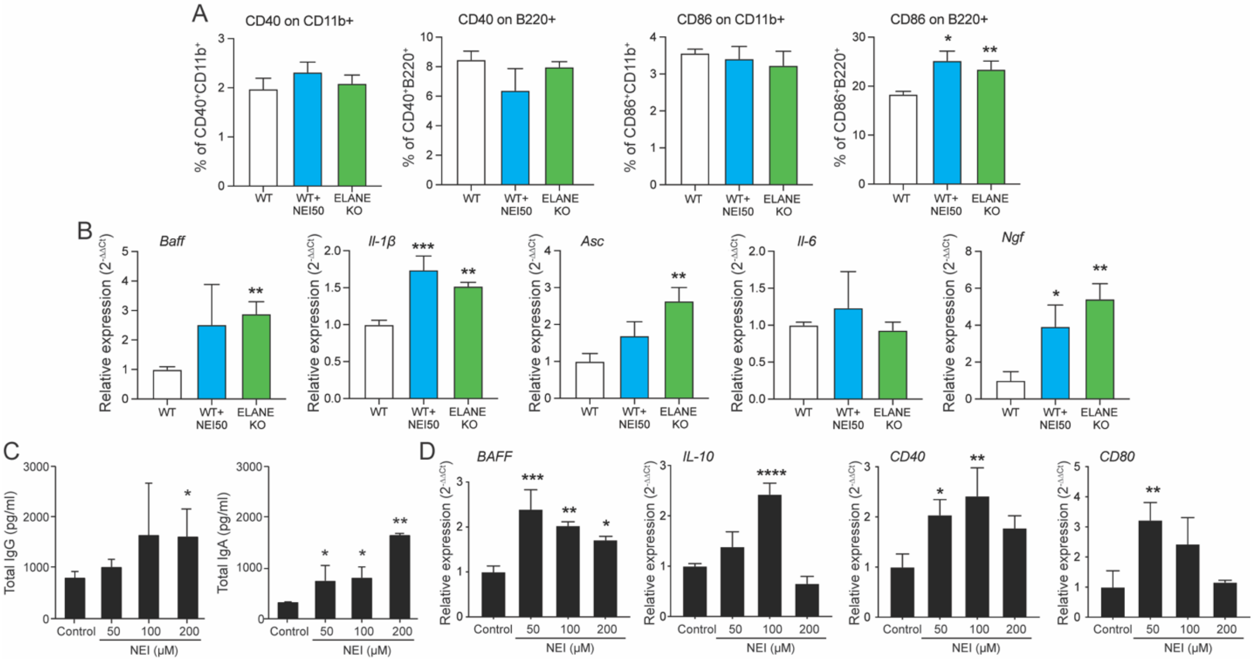

Figure S3. Neutrophil elastase inhibitor or elastase deficiency enhances the activation of B cells and the. expression of genes related with Ig-class switching. (A) Expression of costimulatory molecules from myeloid cells ($CD11b^+$) or B cells ($CD19^+$). Spleen cells were collected from WT and ELANE KO mice and cultured with NEI (50 µM) for 48 hrs. (B) Expression of mRNAs related with Ig-class switching. Spleen cells were collected and cultured with or without NEI (50 µM) for 24 hrs. (C) Neutrophil elastase inhibitor stimulates IgG and IgA production by porcine spleen cells. Cells were cultured for 6 days in the absence or the presence of the NEI. Culture supernatants were then collected and the amount of IgG and IgA secreted was analyzed by ELISA. Data are expressed as mean ± SD. (representative of 2 independent experiments with n=3 piglets/group). (D) Neutrophil elastase inhibitor stimulates expression of cytokines and costimulatory molecules by human PBMC. PBMC were cultured for 48 hrs in the absence (Control) or the presence of NEI. Cytokine and costimulatory molecules mRNA

expression were then analyzed by real-time RT-PCR. Data are expressed as mean ± SD. (representative of 3 independent experiments with n=3/group). $^{*}p < 0.05$, $^{**}p < 0.01$, $^{***}p < 0.001$, $^{****}p < 0.0001$ compared to the control group. $^{\#}p < 0.05$, $^{\#\#}p < 0.01$, $^{\#\#\#}p < 0.001$ compared to Ag+Alum.

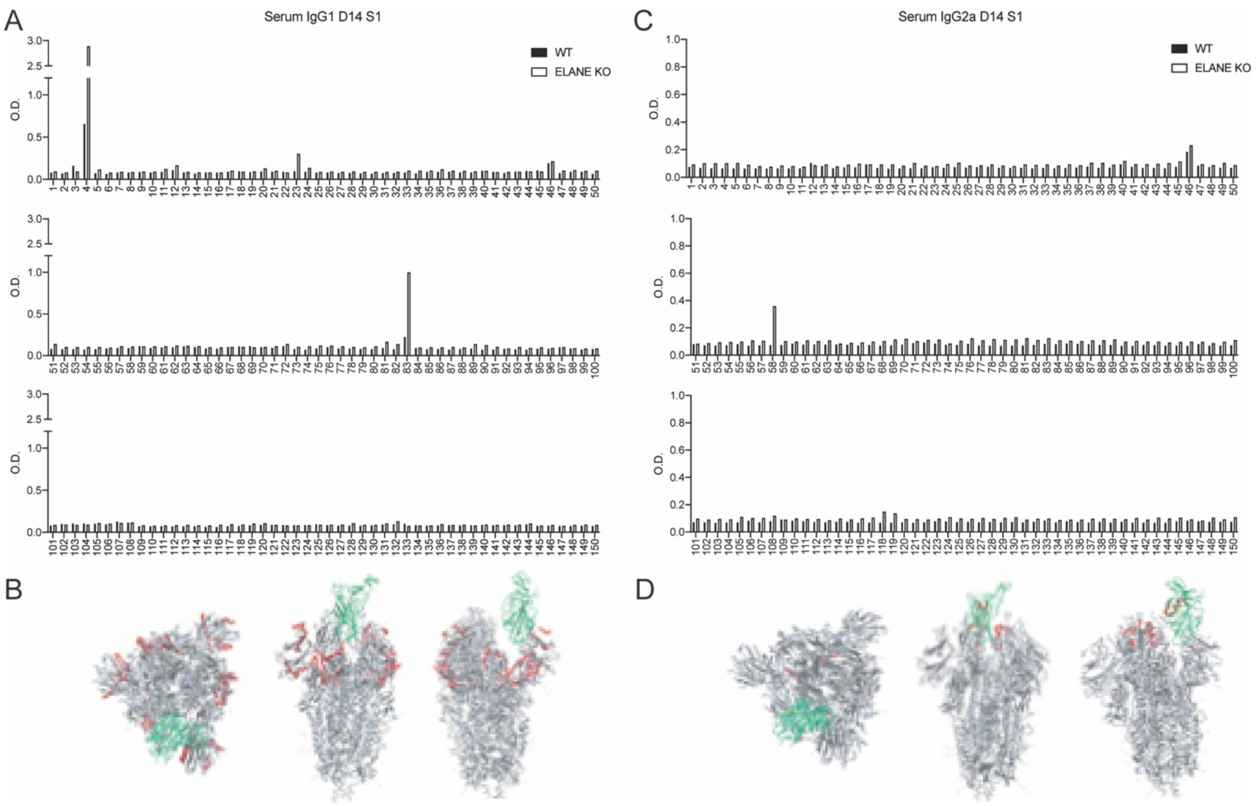

Figure S4. Lack of neutrophil elastase broadens SARS-CoV-2 spike 1 epitope responses to immunization with alum-adsorbed injected vaccine. ELANE KO mice and WT mice were immunized three times, a week apart, by i.p. injection of 100 μl of vaccine containing antigens (10 μg PA + 20 μg S1 of SARS-CoV-2) adsorbed to alum (2 mg) (Ag+Alum). (A and C) Epitope mapping of serum IgG1 (A) and IgG2a(C). Pooled sera of immunized mice at 1:250 dilution and SARS-CoV-2 spike 1-specific IgG1 and IgG2a epitope responses were analyzed by ELISA (B and D). Top and side views of 3D imaging of spike protein and localization of epitopes of IgG1 (B) and IgG2a (D). Epitopes are indicated in red. Receptor binding domain (RBD) is shown in green.
